# Efficacy of the Mental Health App “Intellect” to Improve Body Image and Self-compassion in Young Adults: A Randomized Controlled Trial With a 4-Week Follow-up

**DOI:** 10.2196/41800

**Published:** 2022-11-21

**Authors:** Wen Yi Ong, Oliver Sündermann

**Affiliations:** 1 Department of Psychology National University of Singapore Singapore Singapore; 2 Intellect Singapore Singapore

**Keywords:** body image, body image program, mobile health app, mHealth app, mobile-based interventions, dissonance-based interventions, self-compassion

## Abstract

**Background:**

Body image concerns are prevalent among young adults, who may be vulnerable to developing body image concerns because of particular risk factors associated with this life period. With technological advancements, digital mobile health (mHealth) apps are cost-effective and scalable interventions. Thus, mHealth apps can be explored as a form of prevention effort to alleviate body image concerns in young adults.

**Objective:**

In this randomized controlled trial, we examined the effectiveness of a self-guided mHealth app in improving body image and self-compassion in a sample of university students.

**Methods:**

Participants (N=310) were randomized to a 9-day self-guided body image and self-compassion mHealth app (n=149) and to an active waitlist control group (n=161), where they completed a similarly structured 9-day program on cooperation. Both programs consisted of content learning and activities such as quizzes, with the number and length of activities matched for both programs. Measures were obtained at baseline, upon completion of the programs (after the intervention), and at 4-week follow-up.

**Results:**

The intervention group for female participants reported significant reduction in body dissatisfaction (*P*<.001) and improvements in body appreciation (*P*<.001) and self-compassion (*P*=.001) compared with the active waitlist control group after the intervention. Similarly, for male participants after the intervention, a significant reduction was found in the intervention group in body dissatisfaction (*P*<.001) after the intervention as well as improvements in body appreciation (*P*=.02) and self-compassion (*P*=.047). The effects were maintained at 4-week follow-up for female participants on body dissatisfaction (*P*<.001), body appreciation (*P*<.001), and self-compassion (*P*=.02) but not for male participants. On body image risk factors, significant reductions were found for female participants after the intervention for thin-ideal internalization (*P*<.001), peer pressure (*P*=.002), and media pressure (*P*<.001) after the intervention, while the effects were only maintained for thin-ideal internalization (*P*=.008) and media pressure (*P*=.01) at 4-week follow-up, compared with the active waitlist control group. As for male participants, no intervention effects were found both after the intervention and at follow-up for all body image risk factors of muscularity internalization, peer pressure, and media pressure. Both apps were acceptable and participants engaged equally across the intervention and active waitlist control groups, as indicated on a measure of app engagement (*P*=.76).

**Conclusions:**

This study provides preliminary evidence for a self-guided mHealth app in improving body image concerns and self-compassion in young adult university students. Future studies should include longer follow-ups, and examine its effects with the wider populations of young adults.

**Trial Registration:**

ClinicalTrials.gov NCT04977973; https://clinicaltrials.gov/ct2/show/NCT04977973

## Introduction

### Background

Body image problems are highly prevalent among adolescents and young adults and have been frequently implicated in the development and maintenance of problematic eating behaviors [1,2] and body dysmorphic disorder [3,4]. Young adults may be particularly vulnerable to developing body image problems because of particular risk factors associated with this life period [5,6]. As young people transition through this developmental period, their bodies change in height, weight, and proportion while being exposed to social pressures associated with physical appearances [7]. Sociocultural factors play an important role in the development of body image concerns. According to the tripartite influence model, media, peers, and parents are the main sources of social influence on an individual’s body image [8]. These influences largely take place through appearance-ideal internalization and appearance comparisons [9]. Research has shown that women tend to desire the thin-ideal, whereas men desire muscularity and weight [10]. Appearance-ideal internalization was found to mediate the relationship between sociocultural influences and body dissatisfaction [11,12], with greater internalization exacerbating body dissatisfaction [9]. This suggests that reducing the internalization of appearance ideals likely decreases body dissatisfaction.

Individuals tend to engage in upward appearance comparison, whereby they compare their appearance to others whom they perceive to be more attractive [13]. Consequently, they experience lower body esteem and higher body dissatisfaction, which perpetuate further appearance comparison [9,14].

Exposure to media contributes to appearance-ideal internalization and appearance comparison. Media and social networking platforms are filled with ideal-looking images of the self and others which are often skewed representations of reality [15,16]. Through social learning, individuals tend to normalize such content and internalize them as reality [15,17]. Moreover, individuals with vulnerability factors such as preexisting body image concerns, low self-esteem, depression, perfectionism, or overvalued appearance ideals are more likely to engage in appearance comparison to seek assurance and validation [18]. Altogether, appearance comparison on media platforms contributes to the development and maintenance of body dissatisfaction [9], highlighting the importance of addressing media literacy in reducing body image concerns.

Among peers, appearance-focused comparison and appearance-related conversations and activities may also increase body dissatisfaction as they increase individuals’ awareness of their bodies, strengthen the internalization of appearance ideals, and negatively alter personal attitudes and beliefs in relation to beauty standards [19].

Apart from sociocultural influences, ruminative cognitive styles have been associated with greater body dissatisfaction [20]. Rumination is a response style to distress wherein individuals focus on repetitive thoughts and feelings about the distress [21]. As such, distress arising from negative body image may elicit rumination about one’s appearance, which in turn contributes to body dissatisfaction [20].

### Positive Body Image and Self-compassion

Positive body image is characterized as being accepting, appreciating, and respecting of our bodies through attending to the body’s needs, protecting ourselves against unrealistic body ideals, having broader conceptualizations of beauty, and filtering information in a body-protective manner [22,23]. In qualitative studies, participants with positive body image actively rejected unrealistic media images to protect their body image [24,25]. Growing literature highlights that self-compassion contributes to a positive body image [26]. Self-compassion has been found to buffer the impact of media pressure on thin-ideal internalization in women [23], and to reduce body image distress and body dissatisfaction, reduce rumination, and increase body appreciation [23,27,28]. Homan and Tylka [29] highlighted that women who were high in self-compassion maintained high levels of body appreciation in the face of body-related comparisons. Thus, enhancing individuals’ self-compassion may reduce the effects of negative body image and promote positive body image.

Intervention programs targeting body image risk and protective factors have been developed in the last 2 decades. Psychoeducational and cognitive behavioral programs have been effective in improving body image concerns, reducing disordered eating behaviors and attitudes, thin-ideal internalization, and dieting in adolescents and young adults [30,31]. Dissonance-based interventions are also increasingly adopted to address health and social behaviors [32]. For example, the *Body Project* adopted dissonance-based approaches in a group setting by having participants voluntarily critique and take a counterattitudinal stance against the thin-ideal in verbal, written, and behavioral activities [33]. It was theorized that the discrepancy generated between participants’ personal beliefs (eg, thinner is better) and the counterattitudinal arguments made against pursuing thinness would elicit discomfort, and the discomfort would be alleviated by adjusting their personal beliefs to be more in line with the anti–thin-ideal statements [33]. Efficacy trials of the *Body Project* showed reduced eating disorder risk factors (eg, thin-ideal internalization and body dissatisfaction) and fewer eating disorder symptoms in female adolescents and young adults with body image concerns compared with assessment-only control conditions or alternative interventions, with numerous effects sustained up to 3-year follow-ups [34,35]. The *Body Project M* designed for male participants found that cognitive dissonance approach improved outcomes related to male participants’ dissatisfaction with body fat and muscularity, body appreciation, muscularity-enhancing behaviors, appearance comparison, and internalization after the intervention, with all outcomes except dissatisfaction with muscularity and internalization being sustained at 3-month follow-up [36]. Encouraging findings were also found for the *Body Project: More Than Muscles*, wherein significant reductions were observed for several eating disorder risk factors and muscularity and body fat dissatisfaction in male participants, with some outcomes maintained at the 4-week follow-up [37]. A further extension of the *Body Project*, the *Body Project 4 All*, evaluated the effectiveness of a mixed-sex program which found gains to be sustained over a 6-month follow-up [38]. Meta-analyses confirmed the effectiveness of dissonance-based programs [39,40]. Altogether, these studies suggest that dissonance-based interventions are promising in improving body image concerns in male and female participants.

The direct challenging of the thin-ideal within dissonance-based interventions differs somewhat from a self-compassion approach, which aims to promote greater awareness of adverse outcomes created by the thin-ideal. In response to this awareness, self-compassion interventions engender a mindset that promotes self-kindness and connection with others in the face of body image concerns. In other words, self-compassion approaches seek to alter the way in which individuals cope with the distress associated with negative body image [27,28] rather than changing body image itself.

Self-compassion interventions are gaining empirical support in alleviating body image concerns. Self-compassion meditation and single-session self-compassion writing tasks can reduce women’s body dissatisfaction and body shame and improve self-compassion and body appreciation [27,41]. A recent randomized controlled trial (RCT) by Toole et al [42] found self-compassion and dissonance-based interventions for young women with body image distress to be comparable with and more effective than waitlist control and suggested that integrating both self-compassion and dissonance-based approaches in interventions for body image may increase the acceptability of the interventions and reap more beneficial outcomes.

Self-guided, mobile-based body image programs have been developed and evaluated in light of technological advancements [43-45]. The 7-day mobile app study by Kosinski [44] led to a decrease in participants’ body dissatisfaction, drive for thinness, and increase in self-esteem. Cerea et al [43] adopted a cognitive behavioral training approach with short, daily, cognitive training exercises for 16 days and found that it reduced body dissatisfaction in female university students. Finally, *BodiMojo*, a 6-week program, which involves sending daily intervention messages on body image and self-compassion–related content, increased appearance esteem and self-compassion in adolescents [45]. In a sample of high school and college students, *BodiMojo* improved participants’ body image and self-compassion [45].

### This Study

Emerging adulthood often marks the onset of body image concerns [35]. Presently, most intervention programs are designed for female participants and are conducted in Western populations. The *Body Project* was only recently modified to cater to male participants and mixed-sex groups [36-38]. However, these programs are conducted face-to-face and not on mobile platforms. Evidence is emerging that mobile apps can provide convenient, effective, and cost-friendly mental health interventions [46]. Therefore, this study evaluated the effectiveness of a self-guided mobile health (mHealth) body image app for both female and male participants. The app adopted both cognitive dissonance and self-compassion approaches, covering the following 3 topics: media literacy, appearance comparisons, and self-compassion. These topics were selected because of the robust empirical evidence that has been found for their role as risk and protective factors of body image concerns. The content was adapted from existing evidence-based interventions for body image, eating disorders, and self-compassion [47-51]. We predicted that the intervention would lead to significant improvements on measures of body image and self-compassion after the intervention and 4-week follow-up, compared with an active waitlist control group.

## Methods

### Participants

The sample consisted of 310 female (age: mean 21.12, SD 2.07 years) and male (age: mean 22.68, SD 2.10 years) adults aged between 18 to 30 years, recruited from the department of psychology’s research participant pool and the research recruitment platform of the National University of Singapore. A poster advertisement was uploaded on the respective recruitment platforms, wherein interested students were able to directly access a web-based link to participate in the study. Participants received either course credits or a reimbursement of SGD $ 15 (US $10.63). A power analysis with G*power 3.1 [52] revealed a minimum number of 128 participants, using a moderate effect size as found in relevant mobile-based body image studies [45,53,54]. We aimed for a total of 308 participants to account for a potential attrition of 20% [45].

### Intervention Conditions

#### Body Image Program

This 9-day program adopted cognitive dissonance and self-compassion approaches designed around the following 3 topics: media literacy, appearance comparison, and self-compassion. At the start of each 3-day period, the participants underwent a 5-minute content learning and dissonance-based or self-compassion activity related to the topic (Textbox 1). The dissonance-based activity involved participants challenging sociocultural influences regarding media messages, appearance ideals, and appearance comparison. Participants typed their answers to questions which guided them in challenging sociocultural ideals. Self-compassion interventions involved psychoeducation and experiential activities. Participants were also given a cognitive or behavioral task, which encouraged noticing and challenging sociocultural influences in their daily lives, or practicing self-compassion.

**Figure 1:**
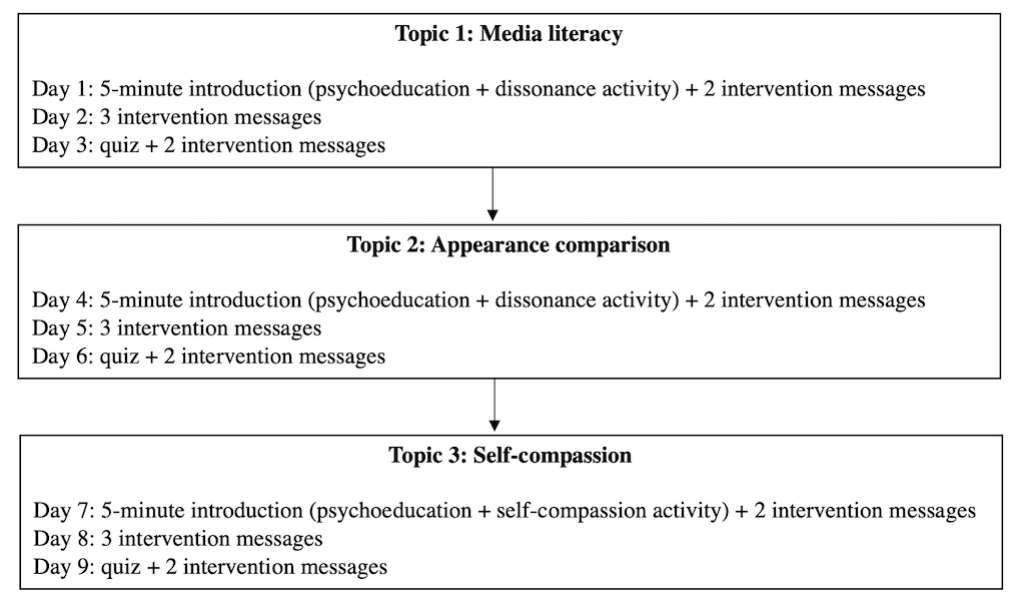
Flow of Body Image Program.

Daily body image and self-compassion–focused messages were sent through the app to participants thrice a day, messages modeled after the *BodiMojo* mobile app (Table 1) [45]. These intervention messages included psychoeducation, affirmations, behavioral tips, short activities, and quizzes to reinforce participants’ learning (Figure 1).

**Overview of the body image program content.**

**Topics and content**

- Topic 1: appearance ideal and media literacy
  Introduce the concepts of body image and appearance ideals
  Highlight how the media influences our appearance ideals and raise awareness of media manipulations
  Elicit discrepancies between participants’ existing beliefs and behaviors about media influences
  Develop participants’ skills in identifying media influences on appearance ideals
- Topic 2: appearance comparison
  Introduce the concept of appearance comparison
  Highlight areas of common appearance comparisons for female and male participants
  Highlight disadvantages and consequences of appearance comparison
  Elicit discrepancies between participants’ existing beliefs and behaviors about appearance ideals and comparison
  Develop participants’ ability to manage situations when appearance comparison arises
- Topic 3: self-compassion
  Introduce the concepts of self-compassion and appearance rumination
  Highlight the disadvantages and consequences of appearance rumination
  Introduce skills to develop participants’ ability to engage in self-compassion to manage negative thoughts and feelings about their body

**Table 1:** Body image and self-compassion–focused intervention messages.

Intervention messages	Examples
Body image	Did you know, media images are often edited after a photoshoot? For example, complexions are cleaned, eyelines are softened, thighs and stomachs are thinned. Hence, it is not wise to match yourself to these appearance ideals. Can you think of other ways in which media images are edited?
Mindfulness	I hope you have not been too hard on yourself this week. Every time you catch yourself being judgmental or critical about yourself or your body Gently acknowledge and hold the thought in your mind. Breathe slowly, and allow yourself to notice the emotional pain or sensations. Remember, it is perfectly ok for your mind to wander. Simply notice it and gently guide your attention back to your body. Slowly, give yourself compassion by reframing the inner dialogue into something encouraging and supportive. Remember, you can always think of how a wise, nurturing friend, parent, teacher or mentor would say to encourage and support you.
Common humanity	Sometimes, our self-critical voice can be so common that we do not even notice when it is present. Many others have felt this way before too. Have you ever noticed what you say to yourself when you are feeling bad about yourself? Let’s try to soften this self-critical voice with compassion.
Self-kindness	Be kind to yourself and your body. Do not say things about your body and self that you would not say to a friend.
Behavioral tips	Try not to check yourself on reflective surfaces when you are up and around! Enjoy your surroundings! :)
Affirmations	Hey! You are a limited edition and one of a kind! Appreciate all the good things that you and your body can do! :)

#### Cooperation Learning Program

Participants in the active waitlist control group engaged in a self-guided learning program on cooperation. The active waitlist control group was chosen instead of a waitlist control as it serves as an attention control to create similar experiences for participants in both groups to control for nonspecific factors that may influence the study outcomes [55]. This 9-day learning program develops participants’ skills to improve group morale and relationships. It consists of content learning once a day and activities such as quizzes, and the number and length of activities were matched to the body image app.

### Ethics Approval

**Figure 2:**
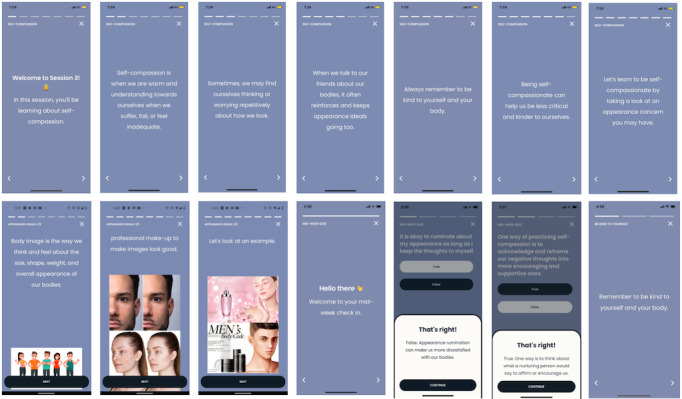
Screenshots of the body image program.

Ethics approval for this study was obtained from the National University of Singapore’s institutional review board (NUS-IRB-2021-85), and it was preregistered with ClinicalTrials.gov (registration number: NCT04977973). The methods and results described complied with the CONSORT (Consolidated Standards of Reporting Trials; 2010) guidelines for reporting RCTs (Figure 2) [56]. Data collection took place in Singapore in an entirely web-based setting.

### Procedure and Participant Flow

Participants first read the Participation Information Sheet on Qualtrics. After providing informed consent, participants completed measures on body image, body image risk factors, and self-compassion to obtain baseline ratings. Thereafter, participants were randomized to 1 of 2 conditions, intervention or active waitlist control, using simple randomization procedures. In this study, blinding of participants was marginally feasible as the content of the intervention programs that the participants engaged in were different in nature. However, participants were not outwardly informed of the real function of each intervention condition or of the real nature of the study being to evaluate the effectiveness of the body image program. The title of the study made known to participants was kept general (*The effectiveness of a self-guided mobile phone application in improving the way we see ourselves and our bodies*) to reduce the demand characteristics of the participants.

Next, the participants downloaded the mobile app and were guided on how to navigate the app. Participants in the intervention group underwent 9 days of body image training, while participants in the active waitlist control group underwent 9 days of the cooperation learning program. The anticipated time participants spent on each program was comparable (<5 minutes per day).

Participants filled out the same questionnaires upon program completion (postintervention measure) and after 4 weeks (follow-up measure). The feedback questionnaire was administered only after the intervention.

After the 6-week data collection period, participants were debriefed about the purpose and real intent of the study. Participants in the active waitlist control group were given access to the body image program.

### Outcome Measures

#### Overview

Body Image Ideals Questionnaire (BIQ [57]) is a 22-item scale that assesses body image satisfaction-dissatisfaction by measuring the degree of congruence or discrepancy in one’s perceived and idealized physical attributes. On a scale ranging from 0 (exactly as I am) to 3 (very unlike me), participants rated the degree to which they resembled their physical ideal on 11 physical attributes. Next, participants rated the importance that they assigned to attaining their ideal on each physical attribute. The cross-products of the discrepancy and importance ratings for each physical attribute were obtained and a composite BIQ score was computed. Higher scores indicated greater disparity between one’s perceived and ideal physical attributes, suggesting higher levels of body dissatisfaction. The BIQ showed good internal consistency, with a Cronbach α of .81 for male participants and .76 for female participants.

The Body Appreciation Scale-2 [58] is a 10-item scale that assesses individuals’ positive attitudes toward their bodies. The items are scored on a scale from 1 (never) to 5 (always). Scores on all items are averaged with higher scores indicating greater body appreciation. The Body Appreciation Scale-2 has excellent internal consistency, with a Cronbach α of .96 for male participants and .97 for female participants.

The Sociocultural Attitudes Toward Appearance Questionnaire-4 Revised [59] measures internalization ideals and appearance-related sociocultural pressures. The 7 subscales consist of 31 items for female participants and 28 items for male participants on a scale ranging from 1 (definitely disagree) to 5 (definitely agree). Higher scores on each subscale indicate higher levels of internalization and sociocultural pressure. In this study, the subscales of Internalisation: Thin/Low Body Fat (for female participants), Internalisation: Muscularity (for male participants), and Pressures: Peers and Media were used. The internal consistencies of the subscales are good, with Cronbach α of ≥.82 in a sample of university female participants and Cronbach of ≥.75 in a sample of university male participants.

Self-Compassion Scale-Short Form [60] is a 12-item scale that measures self-compassion on 6 subscales. Each item is scored from 1 (almost never) to 5 (almost always). A total self-compassion score is the mean of all 6 subscales, with higher scores indicating higher levels of self-compassion. The internal consistency of the scale is excellent, with a Cronbach α of .86.

#### App Engagement

App Engagement Scale [61] is a 7-item scale that measures participants’ engagement on the phone app with scores ranging from 1 (definitely disagree) to 5 (definitely agree). A total score is derived by adding the scores from each item. Internal reliability of the scale is good, with a Cronbach α of .84.

### Analytic Approach

Statistical analyses were conducted using SPSS (version 26.0; IBM Corp). As previous research has found that female and male participants’ body image are dissimilar and that they respond differently to intervention programs [62,63], analyses for this study were conducted separately for female and male participants. Intent-to-treat analyses were conducted to address loss of participant data because of participant withdrawal or technical difficulties, by carrying forward the participants’ last reported score. Independent 2-tailed *t* tests were also conducted to determine if participants who withdrew or could not continue because of technical difficulties differed significantly from those who remained in the study on any demographic and outcome measures. This informed of attrition-related bias, if any. Finally, missing scores on the App Engagement Scale (AES) were substituted using mean substitution [64].

An analysis of covariance (ANCOVA) examined if changes in outcome measures after intervention and at follow-up were significantly different in the intervention group compared with the active waitlist control group. ANCOVA is the recommended analysis for the inferential test of intervention effects [65]. By controlling for baseline scores, any baseline differences that may account for effects in the groups were removed, ensuring that the results after the intervention and at follow-up were because of intervention effects [66]. To compare the intervention and active waitlist control groups after the intervention, ANCOVA was conducted on postintervention scores, with baseline scores of the relevant outcome measures entered as the covariate. The α level was set at *P*<.05. Partial eta squared (*η_p_*^2^) was the effect size reported for ANCOVA, while eta squared (*η*^2^) was the effect size reported for 2-tailed *t* tests and ANOVA.

## Results

### Participant Characteristics

**Figure 3:**
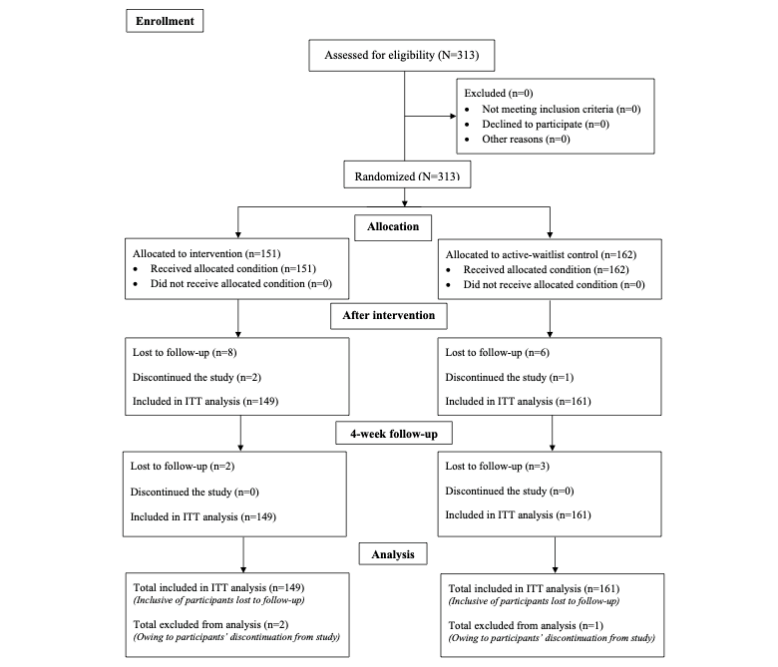
CONSORT (Consolidated Standards of Reporting Trials) flow diagram. ITT: intention-to-treat.

A total of 313 participants completed questionnaires at baseline, 296 (94.57%) participants completed questionnaires after the intervention, and 291 (92.97%) participants completed questionnaires at follow-up (Figure 3).

### Preliminary Analyses

The intervention and active waitlist control groups did not differ significantly on demographic variables and most outcome measures at baseline (Tables 2, 3, and 4). The only outcome measure with a significant baseline difference was BIQ for male participants (*P*=.03). AES rated after the intervention did not differ significantly between female and male participants (*P*=.51) and between the intervention and active waitlist control groups (*P*=.76).

Intent-to-treat analyses were conducted for participants who were lost to follow-up by carrying forward their last reported scores. Missing scores for AES were substituted with the mean score. Independent 2-tailed *t* tests did not reveal any attrition-related biases across demographic and outcome variables (*P*>.10).

**Table 2:** Descriptive statistics for demographic variable of sex by condition.

Demographic variable		Intervention condition (n=149), n (%)	Active waitlist control condition (n=161), n (%)	*P* value	
**Sex**					.32
	Female	98 (65.8)	115 (71)		
	Male	51 (34.2)	46 (29)		

**Table 3:** Descriptive statistics of baseline demographic and outcome variables by condition for female participants (N=213).

Variable		Intervention condition	Active waitlist control condition	*P* value	
Age (years), mean (SD)		21.05 (1.96)	21.18 (2.17)	.65	
BMI (kg/m^2^), mean (SD)		20.20 (2.59)	20.85 (3.32)	.14	
**Race, n (%)**					.13
	Chinese	94 (95.9)	102 (88.7)		
	Malay	0 (0)	1 (0.9)		
	Indian	1 (1)	8 (7)		
	Others	3 (3.1)	4 (3.5)		
BIQ^a^ score, mean (SD)		2.14 (1.21)	2.10 (1.17)	.80	
BAS^b^ score, mean (SD)		3.44 (0.77)	3.50 (0.75)	.54	
SCS-SF^c^ score, mean (SD)		2.88 (0.63)	2.98 (0.55)	.23	
SATAQ-4R^d^: Internalisation Thin/Low Body Fat score, mean (SD)		3.15 (0.86)	3.03 (0.89)	.32	
SATAQ-4R: peer pressure score, mean (SD)		2.23 (1.00)	2.14 (0.98)	.50	
SATAQ-4R: media pressure score, mean (SD)		3.26 (1.04)	3.18 (1.23)	.61	

^a^BIQ: Body Image Ideals Questionnaire.

^b^BAS-2: Body Appreciation Scale-2.

^c^SCS-SF: Self-Compassion Scale-Short Form.

^d^SATAQ-4R: Sociocultural Attitudes Toward Appearance Questionnaire-4 Revised.

**Table 4:** Descriptive statistics of baseline demographic and outcome variables by condition for male participants (N=97).

Variable		Intervention condition	Active waitlist condition	*P* value	
Age (years), mean (SD)		23.06 (2.33)	22.26 (1.76)	.06	
BMI (kg/m^2^), mean (SD)		22.19 (3.59)	22.79 (3.17)	.39	
**Race, n (%)**					.07
	Chinese	50 (98)	39 (84.8)		
	Malay	0 (0)	1 (2.2)		
	Indian	0 (0)	5 (10.9)		
	Others	1 (2)	1 (2.2)		
BIQ^a^ score, mean (SD)		2.12 (1.17)	1.59 (1.12)	.03^b^	
BAS^c^ score, mean (SD)		3.45 (0.73)	3.55 (0.68)	.48	
SCS-SF^d^ score, mean (SD)		3.01 (0.52)	3.05 (0.49)	.67	
SATAQ-4R^e^: muscularity internalization score, mean (SD)		3.44 (0.74)	3.17 (0.82)	.32	
SATAQ-4R: peer pressure score, mean (SD)		2.79 (1.14)	2.78 (1.10)	.94	
SATAQ-4R: media pressure score, mean (SD)		2.70 (1.10)	2.71 (1.05)	.96	

^a^BIQ: Body Image Ideals Questionnaire.

^b^*P*<.05.

^c^BAS-2: Body Appreciation Scale-2.

^d^SCS-SF: Self-Compassion Scale-Short Form.

^e^SATAQ-4R: Sociocultural Attitudes Toward Appearance Questionnaire-4 Revised.

### Main Analyses

#### Overview

Most outcome variables met the assumption tests for ANCOVA. The homogeneity of variance assumption was violated for a small number of outcome measures. However, because of the robustness of ANCOVA when sample sizes in each group are relatively equal [67], analysis using ANCOVA proceeded. The assumption of independence between independent variable and covariate was met for all outcome measures (*P>*.20), except BIQ for male participants. The assumption of homogeneity of regression slopes were violated in 2 variables for female participants (postintervention BIQ; postintervention SATAQ-4R Internalisation: Thin/Low Body Fat). For these variables, 1-way ANOVA was conducted using differences in scores between baseline and after the intervention and baseline and follow-up, respectively.

#### Female Participants

On body image measures, the intervention group reported significantly lower body dissatisfaction, and significantly higher body appreciation, after the intervention and at follow-up, compared with the active waitlist control group. The effect sizes were between moderate to large at both postintervention and follow-up. On thin-ideal internalization, the intervention group reported significantly lower internalization of thin-ideal scores after the intervention and at follow-up, compared with the active waitlist control group, with small to moderate effect sizes after the intervention and at follow-up. Significant score reductions were found on measures of peer and media pressure after the intervention in the intervention group, compared with the active waitlist control group, with small to moderate effect sizes. At follow-up, a significant difference was only found for reduction in media pressure, and no significant difference was found for peer pressure. Finally, significant differences were found for the self-compassion measure after the intervention and at follow-up, compared with the active waitlist control group (Table 5).

**Table 5:** Means (SDs), univariate F test values, and effect sizes for outcome variables in female participants.

Variable	Scale range	Baseline, mean (SD)		After the intervention						Follow-up				
		Intervention	Control	Intervention, mean (SD)	Control, mean (SD)	*F* test (*df*)	*P* value	Effect size^a^	Intervention, mean (SD)		Control, mean (SD)	*F* test (*df*)	*P* value	Effect size^a^
Body Image Ideals Questionnairescore^b^	−3 to 9	2.14 (1.21)	2.10 (1.17)	1.30 (0.99)	1.94 (1.20)	26.02^c^ (1)	*<.001^d^*	0.11	1.42 (0.09)		1.91 (0.087)	17.48^c^ (1)	*<.001*	0.077
Body Appreciation Scale-2 score^e^	1 to 5	3.44 (0.77)	3.50 (0.75)	3.79 (0.48)	3.57 (0.44)	37.80^c^ (1)	*<.001*	0.27	3.73 (0.05)		3.60 (0.045)	38.00^c^ (1)	*<.001*	0.27
Self-Compassion Scale-Short Form score^e^	1 to 5	2.88 (0.63)	2.98 (0.55)	3.28 (0.04)	3.09 (0.041)	10.82^f^ (1)	*.001*	0.049	3.20 (0.47)		3.05 (0.043)	5.92^g^ (1)	*.02*	0.027
SATAQ-4R^h^: Internalisation Thin/Low Body Fat^b^ score	1 to 5	3.15 (0.86)	3.03 (0.89)	2.77 (0.85)	3.03 (0.92)	18.49^c^ (1)	*<.001*	0.081	2.79 (0.06)		3.00 (0.053)	7.21^f^ (1)	*.008*	0.033
SATAQ-4R: peer pressure^b^ score	1 to 5	2.23 (1.00)	2.14 (0.98)	2.00 (0.07)	2.28 (0.065)	9.73^f^ (1)	*.002*	0.044	2.12 (0.08)		2.30 (0.073)	2.93 (1)	.09	0.014
SATAQ-4R: Media pressure^b^ score	1 to 5	3.26 (1.04)	3.18 (1.23)	2.77 (0.09)	3.27 (0.080)	18.08^c^ (1)	*<.001*	0.079	2.80 (0.09)		3.12 (0.085)	6.49^a^ (1)	*.01*	0.031

^a^Effect sizes of 0.01=small, 0.06=moderate, and 0.14=large [68].

^b^Lower scores are more desirable.

^c^*P*<.001.

^d^Italicized values indicate a significant *P* value at .05.

^e^Higher scores are more desirable.

^f^*P*<.01.

^g^*P*<.05.

^h^SATAQ-4R: Sociocultural Attitudes Toward Appearance Questionnaire-4 Revised.

#### Male Participants

Male participants in the intervention group reported significantly lower scores on body dissatisfaction after the intervention, compared with the active waitlist control group, with a large effect size. Male participants in the intervention group also reported significantly higher scores for body appreciation and self-compassion after the intervention, compared with the active waitlist control group, with effect sizes ranging from small to moderate. No intervention effects were found after the intervention for muscularity internalization, peer pressure, and media pressure, and at follow-up for all measures (Table 6).

**Table 6:** Means (SDs), univariate F values, and effect sizes for outcome variables in male participants.

Variable	Scale range	Baseline, mean (SD)		After the intervention						Follow-up				
		Intervention	Control	Intervention, mean (SD)	Control, mean (SD)	*F* test (*df*)	*P* value	Effect size^a^	Intervention, mean (SD)		Control, mean (SD)	*F* test (*df*)	*P* value	Effect size^a^
Body Image Ideals Questionnaire^b^	−3 to 9	2.12 (1.17)	1.59 (1.12)	1.24 (0.95)	1.53 (1.20)	16.07^c^ (1)	*<.001^d^*	0.15	1.69 (1.23)		1.32 (1.24)	0.69 (1)	.41	0.007
Body Appreciation Scale-2^e^	1 to 5	3.45 (0.73)	3.55 (0.68)	3.83 (0.065)	3.61 (0.068)	5.71^f^ (1)	*.02*	0.057	3.75 (0.07)		3.60 (0.073)	20.32 (1)	.13	0.024
Self-Compassion Scale-Short Form^e^	1 to 5	3.00 (0.60)	3.15 (0.64)	3.33 (0.054)	3.17 (0.057)	4.039^f^ (1)	*.047*	0.041	3.24 (0.06)		3.12 (0.060)	10.79 (1)	.18	0.019
SATAQ-4R^g^: Muscularity internalisation^b^	1 to 5	3.44 (0.74)	3.28 (0.81)	3.17 (0.092)	3.20 (0.097)	.070 (1)	.79	0.001	3.27 (0.09)		3.25 (0.093)	0.018 (1)	.89	0.000
SATAQ-4R: Peer pressure^b^	1 to 5	2.79 (1.14)	2.78 (1.10)	2.57 (0.12)	2.85 (0.12)	2.72 (1)	.10	0.028	2.48 (0.10)		2.63 (0.11)	0.99 (1)	.32	0.01
SATAQ-4R: Media pressure^b^	1 to 5	2.70 (1.10)	2.71 (1.05)	2.54 (0.13)	2.62 (0.13)	0.17 (1)	.68	0.002	2.78 (0.12)		2.65 (0.12)	0.61 (1)	.44	0.006

^a^Effect sizes of 0.01=small, 0.06=moderate, and 0.14=large [68].

^b^Lower scores are more desirable.

^c^*P*<.001.

^d^Italicized values indicate a significant *P* value at .05.

^e^Higher scores are more desirable.

^f^*P*<.05.

^g^SATAQ-4R: Sociocultural Attitudes Toward Appearance Questionnaire-4 Revised.

## Discussion

### Principal Findings

This RCT evaluated the effectiveness of a self-guided mHealth app in improving body image and self-compassion in a sample of Asian university students. Our study extended the findings of previous studies by showing that cognitive dissonance and self-compassion approaches delivered on a mobile-based platform can be beneficial in improving body image and self-compassion in young adults.

Our hypotheses for female participants were largely supported. Except for peer pressure whereby intervention effects were not found at follow-up, the intervention group reported significant improvements on body image, body image risk factors, and self-compassion at both postintervention and follow-up, compared with the active waitlist control group. Consistent with past research on longer web-based or face-to-face interventions, our findings showed that a 9-day mobile-based program using cognitive dissonance and self-compassion approaches can reduce body dissatisfaction and its risk factors, improve body appreciation, and improve self-compassion after the intervention and at follow-up in female adults [33,34,42,43,44,51,69]. The moderate to large effect sizes for improvements found on body image and risk factor measures in our body image program are comparable with the average effect sizes found in *eBody Project* after the intervention [54]. Furthermore, our study extended the study by Toole et al [42] by demonstrating that integrating dissonance-based and self-compassion approaches and conducting the intervention on a mobile-based app can be beneficial for body image interventions. Comparable with the study by Toole et al [42], the effect sizes for female participants in our study were also moderate to large for body dissatisfaction, body appreciation, and thin-ideal internalization after the intervention.

Our hypotheses for male participants were partially supported. Unlike findings from *Body 4 All* and *Body Project M,* which found significant improvements in male participants on body satisfaction, body appreciation, dissatisfaction with fat and muscularity, appearance comparison, and internalization of cultural appearance ideals, with some effects sustained at their respective follow-ups [37,38], our study only revealed significantly lower body dissatisfaction, higher body appreciation, and higher self-compassion in the intervention group after the intervention relative to the active waitlist control group. In particular, the effect size for body dissatisfaction was large. Although the results for internalization of muscularity (*P*=.79 and *P*=.89), peer pressure (*P*=.10 and *P*=.32), and media pressure (*P*=.68 and *P*=.44) did not reach statistical significance after intervention and at follow-up, there was a trend observed toward male participants in the intervention group reporting lower scores of muscularity internalization and media and peer pressure after the intervention.

Overall, our findings provide preliminary support for the use of cognitive dissonance and self-compassion approaches on an mHealth app to reduce body image concerns and improve self-compassion in students. On the basis of the cognitive dissonance theory and the dual pathway model [33,70], guiding participants to challenge ideal appearances likely led to the participants’ reduced subscription to appearance ideals, which decreased body dissatisfaction. The 4-week follow-up effects found in female participants for all measures except for peer pressure were encouraging. In particular, the large effect sizes maintained for female participants on the improvements on both body dissatisfaction and body appreciation both after the intervention and at the 4-week follow-up are noteworthy. In addition to the use of cognitive dissonance techniques in our body image program to challenge appearance ideals directly, the integration of self-compassion components may have contributed to female participants’ enhanced self-awareness of appearance ideals, elicited a compassionate view of themselves and their bodies, and helped them to cope with the body image distress by fostering self-kindness and connection with others. Altogether, these may have translated to greater intrinsic self-worth and enhanced acceptance and appreciation of their bodies [27,42]. In addition, a self-compassion approach for the body image program may have been more appealing for young female adults, and increased their acceptance of the program [42].

Consistent with *BodiMojo’s* 6-week mobile-based intervention, our 9-day mobile-based intervention revealed comparable small to moderate effect sizes in self-compassion after the intervention for female and male participants. Compared with *BodiMojo* and the face-to-face programs of the *Body Project*, our study found similar or larger effect sizes for body image in both sexes after the intervention [36,45,71]. These suggest that beneficial effects for body image and self-compassion can be obtained much faster than through longer web-based or face-to-face programs in reducing body image concerns and improving self-compassion in young adults. Although longer programs may provide more opportunities for users to learn and practice skills to elicit behavior change [72], briefer interventions could also be of value in sustaining users’ engagement and reducing attrition rates.

Although significant improvements in self-compassion were found for male participants after the intervention, the effect was weaker, and not significant at follow-up. As self-compassion has been identified as a crucial factor in reducing body dissatisfaction and enhancing body appreciation in female and male participants [29,73,74], the weaker effects observed for self-compassion in male participants may have had a downstream effect and explained the lack of follow-up effects for male participants’ body dissatisfaction and body appreciation.

Sex differences in self-compassion may explain the lack of follow-up effects for self-compassion in male participants. A meta-analysis conducted by Yarnell et al [75] suggested that self-compassion approaches may be more effective for women than men, as feminine gender role norms associated with nurturance, self-sacrifice, and caregiving may facilitate the fostering of compassion toward the self. On the contrary, masculine gender norms, which tend to be associated with being strong, unemotional, pragmatic, and independent may form barriers to men being tender and caring toward themselves in times of need [76,77]. Hence, men with higher masculine norm conformity may find it challenging to acquire self-compassion [78]. Nonetheless, individuals may not necessarily conform to traditional gender role orientations [78], and our study did not explore participants’ conformity to gender norms. As literature in this area remains relatively new, further research is required to improve our understanding of gender role norms in self-compassion, to effectively tailor self-compassion approaches for female and male participants.

Several reasons are conceivable why peer pressure did not reveal differential effects for male participants and for female participants at follow-up. During this developmental phase as a young adult, interpersonal relationships are crucial for female and male participants [79,80]. Female participants tend to experience greater sensitivity and stress because of interpersonal rejection [81,82]. Coupled with female participants’ tendency to associate body image with the perception of peer acceptance and the lack of effective coping strategies, female participants may have found it challenging to manage peer pressures [83,84]. Thus, equipping female participants with stress and communication management skills may be a more targeted approach for enhancing female participants’ capacity to reduce peer pressures [84]. The lack of significant findings for peer pressure in female participants at follow-up may also be because of a floor effect, as participants in both intervention and active waitlist control groups had low ratings on the measure at baseline, thus limiting intervention effects at follow-up. As for male participants, studies found that greater body dissatisfaction was related to peer stressors focused on personality characteristics or achievements [81,84]. Thus, targeting body image and its risk factors may be less effective in reducing peer pressures for male participants.

The lack of significant reduction in the internalization of muscularity after the intervention and at follow-up may be accounted for by at least 2 possible reasons. First, adaptations made to our mixed-sex body image program to incorporate male body image concerns may be insufficient in addressing muscular-ideal internalization in a targeted manner. For example, the terminology being changed to “ideal appearances” and insufficient examples for male participants may have led to an inadequate understanding of concerns related to muscular-ideal internalization, thus watering down the intervention effects for male participants. Second, male participants may be less engaged in the body image program than female participants, because of the lack of masculine points of reference [85]. Although app engagement ratings did not differ between sexes, there may be socially desirable or careless responses on the self-report questionnaires. This is also supported by research which highlighted that male participants are less likely to engage in body image interventions and digital mental health apps than female participants [86,87]. Moreover, as participants’ responses on the app program were not accessible by researchers to protect users’ confidentiality, the quality and length of the counterattitudinal written responses could not be objectively ascertained. Thus, there is a possibility that the men’s lower app engagement may have weakened the intervention effects.

Finally, the lack of group differences on media pressure for male participants was unexpected, as media literacy was previously found to be effective in reducing pressures from media influences [88]. A possible explanation may be that our media literacy content was inclined toward female appearance ideals and risk factors and thus the lack of specificity and relevance to male appearance ideals and risk factors may have reduced its effectiveness for male participants.

### Strengths and Limitations

First, some causal conclusions can be drawn [89]. The use of an active waitlist control group as an attention waitlist control allowed us to disentangle the effects of attention and other nonspecific factors from the intervention effects, thus strengthening the RCT’s effects [90]. Another strength was the low attrition rate. Although some participants withdrew or could not continue because of technical glitches, the overall attrition rate was <10%, with no attrition-related biases. This conferred greater strength to the overall validity of our study. Separate analyses conducted for female and male participants in light of sex differences in body image allowed us to identify that our mixed-sex program was less effective for male participants than for female participants and thus identify ways to enhance intervention effects for male participants. Finally, the 4-week follow-up period gives some confidence that effects for female participants can be maintained over a short-term period.

This study has some limitations. First, student participants may limit the generalizability of findings, as university students and the public may differ in factors such as level of education [91]. Hence, the university sample may not be representative of the general young adult population. Secondly, self-report measures are prone to social desirability bias, expectancies, and demand characteristics, which all may have contributed to the observed effects. Third, incentivizing participants with course credits or money may have motivated their participation and retention.

The sample size for male participants in our study was small despite additional recruitment efforts and thus was likely underpowered. Moreover, in view of sex differences in body image, more research is required to better understand ways to increase the effectiveness of mobile-based body image programs for male participants in a mixed-sex format and address muscularity concerns. To capture male participants’ body image concerns more accurately, male-specific measures such as Male Body Attitudes Scale [92] can be used in future studies.

Finally, in light of increasing studies which found self-compassion to mediate the effects of body image interventions, future studies can examine self-compassion as a mechanism of change. It would also be beneficial to obtain qualitative feedback from participants on elements of the body image program, such as their perception of the tone or number of intervention messages, to evaluate the effectiveness of the program.

### Conclusions

Overall, this RCT provides preliminary but encouraging support for the effectiveness of a self-guided mHealth body image app using cognitive dissonance and self-compassion approaches for university students. Mobile-based well-being programs are cost-effective and accessible and can thus be widely disseminated to benefit the masses. Future research should seek to further enhance the program’s effectiveness with the wider young adult population.

## Appendix

Multimedia Appendix 1 CONSORT-eHEALTH checklist (V 1.6.1).

## Abbreviations

AES: App Engagement Scale
ANCOVA: analysis of covariance
BIQ: Body Image Ideals Questionnaire
CONSORT: Consolidated Standards of Reporting Trials
mHealth: mobile health
RCT: randomized controlled trial
