# Supplementary material for: Efficacy of the Mental Health App “Intellect” to Improve Body Image and Self-compassion in Young Adults: A Randomized Controlled Trial With a 4-Week Follow-up
Source: JMIR Mhealth Uhealth. 2022 Nov 21;10(11):e41800. doi: 10.2196/41800 (PMC9723978; doi:10.2196/41800)
Supplement: Multimedia Appendix 1 [file mhealth_v10i11e41800_app1.pdf]

CONSORT-EHEALTH (V 1.6.1) - Submission/Publication Form

The CONSORT-EHEALTH checklist is intended for authors of randomized trials evaluating web-based and Internet-based applications/interventions, including mobile interventions, electronic games (incl multiplayer games), social media, certain telehealth applications, and other interactive and/or networked electronic applications. Some of the items (e.g. all subitems under item 5 - description of the intervention) may also be applicable for other study designs.

The goal of the CONSORT EHEALTH checklist and guideline is to be
a) a guide for reporting for authors of RCTs,
b) to form a basis for appraisal of an ehealth trial (in terms of validity)

CONSORT-EHEALTH items/subitems are MANDATORY reporting items for studies published in the Journal of Medical Internet Research and other journals / scientific societies endorsing the checklist.

Items numbered 1., 2., 3., 4a., 4b etc are original CONSORT or CONSORT-NPT (non-pharmacologic treatment) items.
Items with Roman numerals (i., ii, iii, iv etc.) are CONSORT-EHEALTH extensions/clarifications.

As the CONSORT-EHEALTH checklist is still considered in a formative stage, we would ask that you also RATE ON A SCALE OF 1-5 how important/useful you feel each item is FOR THE PURPOSE OF THE CHECKLIST and reporting guideline (optional).

Mandatory reporting items are marked with a red *.
In the textboxes, either copy & paste the relevant sections from your manuscript into this form - please include any quotes from your manuscript in QUOTATION MARKS,
or answer directly by providing additional information not in the manuscript, or elaborating on why the item was not relevant for this study.

YOUR ANSWERS WILL BE PUBLISHED AS A SUPPLEMENTARY FILE TO YOUR PUBLICATION IN JMIR AND ARE CONSIDERED PART OF YOUR PUBLICATION (IF ACCEPTED).
Please fill in these questions diligently. Information will not be copyedited, so please use proper spelling and grammar, use correct capitalization, and avoid abbreviations.

DO NOT FORGET TO SAVE AS PDF _AND_ CLICK THE SUBMIT BUTTON SO YOUR ANSWERS ARE IN OUR DATABASE !!!

Citation Suggestion (if you append the pdf as Appendix we suggest to cite this paper in the caption):
Eysenbach G, CONSORT-EHEALTH Group
CONSORT-EHEALTH: Improving and Standardizing Evaluation Reports of Web-based and Mobile Health Interventions
J Med Internet Res 2011;13(4):e126
URL: http://www.jmir.org/2011/4/e126/

Your response is too large. Try shortening some answers.

wen-y-i@hotmail.com (not shared) Switch account

Draft not saved

* Required

Your name *

First Last

Wen Yi Ong

Primary Affiliation (short), City, Country *

University of Toronto, Toronto, Canada

Singapore

Your e-mail address *

abc@gmail.com

wen_yi_@u.nus.edu

Title of your manuscript *

Provide the (draft) title of your manuscript.

A 9-Day Mobile Health App For Improving Body Image and Self-compassion in Young Adults: Randomized Controlled Trial With a 4-Week Follow-up

Name of your App/Software/Intervention *

If there is a short and a long/alternate name, write the short name first and add the long name in brackets.

Body Image program

Your response is too large. Try shortening some answers.

Evaluated Version (if any)

e.g. "V1", "Release 2017-03-01", "Version 2.0.27913"

Your answer

Language(s) *

What language is the intervention/app in? If multiple languages are available, separate by comma (e.g. "English, French")

English

URL of your Intervention Website or App

e.g. a direct link to the mobile app on app in appstore (itunes, Google Play), or URL of the website. If the intervention is a DVD or hardware, you can also link to an Amazon page.

https://play.google.com/store/apps/details?id=co.intellect.app

URL of an image/screenshot (optional)

Your answer

Must be a valid URL

Accessibility *

Can an enduser access the intervention presently?

- ○ access is free and open
- ◉ access only for special usergroups, not open
- ○ access is open to everyone, but requires payment/subscription/in-app purchases
- ○ app/intervention no longer accessible
- ○ Other:

Your response is too large. Try shortening some answers.

Primary Medical Indication/Disease/Condition *

e.g. "Stress", "Diabetes", or define the target group in brackets after the condition, e.g. "Autism (Parents of children with)", "Alzheimers (Informal Caregivers of)"

Body image concerns

Primary Outcomes measured in trial *

comma-separated list of primary outcomes reported in the trial

Body Image Ideals Questionnaire (BIQ), Body A

Secondary/other outcomes

Are there any other outcomes the intervention is expected to affect?

App Engagement Scale (AES)

Recommended "Dose" *

What do the instructions for users say on how often the app should be used?

- (●) Approximately Daily
- ( ) Approximately Weekly
- ( ) Approximately Monthly
- ( ) Approximately Yearly
- ( ) "as needed"
- ( ) Other:

Your response is too large. Try shortening some answers.

Approx. Percentage of Users (starters) still using the app as recommended after 3 months *

- ( ) unknown / not evaluated
- ( ) 0-10%
- ( ) 11-20%
- ( ) 21-30%
- ( ) 31-40%
- ( ) 41-50%
- ( ) 51-60%
- ( ) 61-70%
- ( ) 71%-80%
- ( ) 81-90%
- ( ) 91-100%
- (x) Other: App program ends after 9 days

Overall, was the app/intervention effective? *

- ( ) yes: all primary outcomes were significantly better in intervention group vs control
- (x) partly: SOME primary outcomes were significantly better in intervention group vs control
- ( ) no statistically significant difference between control and intervention
- ( ) potentially harmful: control was significantly better than intervention in one or more outcomes
- ( ) inconclusive: more research is needed
- ( ) Other:

Your response is too large. Try shortening some answers.

Article Preparation Status/Stage *

At which stage in your article preparation are you currently (at the time you fill in this form)

- ◯ not submitted yet - in early draft status
- ◯ not submitted yet - in late draft status, just before submission
- ◯ submitted to a journal but not reviewed yet
- ◯ submitted to a journal and after receiving initial reviewer comments
- ◉ submitted to a journal and accepted, but not published yet
- ◯ published
- ◯ Other:

Journal *

If you already know where you will submit this paper (or if it is already submitted), please provide the journal name (if it is not JMIR, provide the journal name under "other")

- ◯ not submitted yet / unclear where I will submit this
- ◯ Journal of Medical Internet Research (JMIR)
- ◉ JMIR mHealth and UHealth
- ◯ JMIR Serious Games
- ◯ JMIR Mental Health
- ◯ JMIR Public Health
- ◯ JMIR Formative Research
- ◯ Other JMIR sister journal
- ◯ Other:

Is this a full powered effectiveness trial or a pilot/feasibility trial? *

- ◯ Pilot/feasibility
- ◉ Fully powered

Your response is too large. Try shortening some answers.

Manuscript tracking number *

If this is a JMIR submission, please provide the manuscript tracking number under "other" (The ms tracking number can be found in the submission acknowledgement email, or when you login as author in JMIR. If the paper is already published in JMIR, then the ms tracking number is the four-digit number at the end of the DOI, to be found at the bottom of each published article in JMIR)

- ○ no ms number (yet) / not (yet) submitted to / published in JMIR
- ◉ Other: ms#41800

TITLE AND ABSTRACT

1a) TITLE: Identification as a randomized trial in the title

1a) Does your paper address CONSORT item 1a? *

I.e does the title contain the phrase "Randomized Controlled Trial"? (if not, explain the reason under "other")

- ◉ yes
- ○ Other:

1a-i) Identify the mode of delivery in the title

Identify the mode of delivery. Preferably use “web-based” and/or “mobile” and/or “electronic game” in the title. Avoid ambiguous terms like “online”, “virtual”, “interactive”. Use “Internet-based” only if Intervention includes non-web-based Internet components (e.g. email), use “computer-based” or “electronic” only if offline products are used. Use “virtual” only in the context of “virtual reality” (3-D worlds). Use “online” only in the context of “online support groups”. Complement or substitute product names with broader terms for the class of products (such as “mobile” or “smart phone” instead of “iphone”), especially if the application runs on different platforms.

	1	2	3	4	5	
subitem not at all important	○	○	○	○	◉	essential

Clear selection

Your response is too large. Try shortening some answers.

Does your paper address subitem 1a-i? *

Copy and paste relevant sections from manuscript title (include quotes in quotation marks "like this" to indicate direct quotes from your manuscript), or elaborate on this item by providing additional information not in the ms, or briefly explain why the item is not applicable/relevant for your study

Yes "A 9-Day "Mobile Health App" For Improving Body Image and Self-compassion in Young Adults: Randomized Controlled Trial With a 4-Week Follow-up"

1a-ii) Non-web-based components or important co-interventions in title

Mention non-web-based components or important co-interventions in title, if any (e.g., “with telephone support”).

	1	2	3	4	5	
subitem not at all important	○	○	●	○	○	essential

Clear selection

Does your paper address subitem 1a-ii?

Copy and paste relevant sections from manuscript title (include quotes in quotation marks "like this" to indicate direct quotes from your manuscript), or elaborate on this item by providing additional information not in the ms, or briefly explain why the item is not applicable/relevant for your study

Item is not applicable as our study is evaluating a mobile app program, all components are mobile app based

1a-iii) Primary condition or target group in the title

Mention primary condition or target group in the title, if any (e.g., “for children with Type I Diabetes”) Example: A Web-based and Mobile Intervention with Telephone Support for Children with Type I Diabetes: Randomized Controlled Trial

	1	2	3	4	5	
subitem not at all important	○	○	○	○	●	essential

Clear selection

Your response is too large. Try shortening some answers.

Does your paper address subitem 1a-iii? *

Copy and paste relevant sections from manuscript title (include quotes in quotation marks "like this" to indicate direct quotes from your manuscript), or elaborate on this item by providing additional information not in the ms, or briefly explain why the item is not applicable/relevant for your study

Yes "A 9-Day Mobile Health App For Improving Body Image and Self-compassion in Young Adults: Randomized Controlled Trial With a 4-Week Follow-up"

1b) ABSTRACT: Structured summary of trial design, methods, results, and conclusions

NPT extension: Description of experimental treatment, comparator, care providers, centers, and blinding status.

1b-i) Key features/functionalities/components of the intervention and comparator in the METHODS section of the ABSTRACT

Mention key features/functionalities/components of the intervention and comparator in the abstract. If possible, also mention theories and principles used for designing the site. Keep in mind the needs of systematic reviewers and indexers by including important synonyms. (Note: Only report in the abstract what the main paper is reporting. If this information is missing from the main body of text, consider adding it)

	1	2	3	4	5	
subitem not at all important	○	○	○	○	◉	essential

Clear selection

Does your paper address subitem 1b-i? *

Copy and paste relevant sections from the manuscript abstract (include quotes in quotation marks "like this" to indicate direct quotes from your manuscript), or elaborate on this item by providing additional information not in the ms, or briefly explain why the item is not applicable/relevant for your study

Yes. "Methods: Participants (N=310) were randomized to a 9-day self-guided body image and self-compassion mHealth app (n=149) and to an active waitlist control group (n=161), where they completed a similarly structured 9-day program on cooperation. Both programs consisted of content learning and activities such as quizzes, with the number and length of activities matched for both programs. Measures were obtained at baseline, upon completion of the programs (after the intervention), and at 4-week follow-up."

Your response is too large. Try shortening some answers.

1b-ii) Level of human involvement in the METHODS section of the ABSTRACT

Clarify the level of human involvement in the abstract, e.g., use phrases like “fully automated” vs. “therapist/nurse/care provider/physician-assisted” (mention number and expertise of providers involved, if any). (Note: Only report in the abstract what the main paper is reporting. If this information is missing from the main body of text, consider adding it)

	1	2	3	4	5	
subitem not at all important	○	○	○	○	◉	essential

Clear selection

Does your paper address subitem 1b-ii?

Copy and paste relevant sections from the manuscript abstract (include quotes in quotation marks "like this" to indicate direct quotes from your manuscript), or elaborate on this item by providing additional information not in the ms, or briefly explain why the item is not applicable/relevant for your study

Yes "Methods: Participants (N=310) were randomized to a 9-day self-guided body image and self-compassion mHealth app (n=149) and to an active waitlist control group (n=161), where they completed a similarly structured 9-day program on cooperation."

1b-iii) Open vs. closed, web-based (self-assessment) vs. face-to-face assessments in the METHODS section of the ABSTRACT

Mention how participants were recruited (online vs. offline), e.g., from an open access website or from a clinic or a closed online user group (closed usergroup trial), and clarify if this was a purely web-based trial, or there were face-to-face components (as part of the intervention or for assessment). Clearly say if outcomes were self-assessed through questionnaires (as common in web-based trials). Note: In traditional offline trials, an open trial (open-label trial) is a type of clinical trial in which both the researchers and participants know which treatment is being administered. To avoid confusion, use “blinded” or “unblinded” to indicated the level of blinding instead of “open”, as “open” in web-based trials usually refers to “open access” (i.e. participants can self-enrol). (Note: Only report in the abstract what the main paper is reporting. If this information is missing from the main body of text, consider adding it)

	1	2	3	4	5	
subitem not at all important	○	○	○	○	◉	essential

Your response is too large. Try shortening some answers.

Does your paper address subitem 1b-iii?

Copy and paste relevant sections from the manuscript abstract (include quotes in quotation marks "like this" to indicate direct quotes from your manuscript), or elaborate on this item by providing additional information not in the ms, or briefly explain why the item is not applicable/relevant for your study

Unable to include in the current abstract due to word count limitations

1b-iv) RESULTS section in abstract must contain use data

Report number of participants enrolled/assessed in each group, the use/uptake of the intervention (e.g., attrition/adherence metrics, use over time, number of logins etc.), in addition to primary/secondary outcomes. (Note: Only report in the abstract what the main paper is reporting. If this information is missing from the main body of text, consider adding it)

	1	2	3	4	5	
subitem not at all important	○	○	○	○	●	essential

Clear selection

Does your paper address subitem 1b-iv?

Copy and paste relevant sections from the manuscript abstract (include quotes in quotation marks "like this" to indicate direct quotes from your manuscript), or elaborate on this item by providing additional information not in the ms, or briefly explain why the item is not applicable/relevant for your study

Unable to include in the current abstract due to word count limitations

Your response is too large. Try shortening some answers.

1b-v) CONCLUSIONS/DISCUSSION in abstract for negative trials

Conclusions/Discussions in abstract for negative trials: Discuss the primary outcome - if the trial is negative (primary outcome not changed), and the intervention was not used, discuss whether negative results are attributable to lack of uptake and discuss reasons. (Note: Only report in the abstract what the main paper is reporting. If this information is missing from the main body of text, consider adding it)

	1	2	3	4	5	
subitem not at all important	○	○	○	○	◉	essential

Clear selection

Does your paper address subitem 1b-v?

Copy and paste relevant sections from the manuscript abstract (include quotes in quotation marks "like this" to indicate direct quotes from your manuscript), or elaborate on this item by providing additional information not in the ms, or briefly explain why the item is not applicable/relevant for your study

Brief summary of conclusion was included in abstract; unable to elaborate on more details in the current abstract due to word count limitations

INTRODUCTION

2a) In INTRODUCTION: Scientific background and explanation of rationale

2a-i) Problem and the type of system/solution

Describe the problem and the type of system/solution that is object of the study: intended as stand-alone intervention vs. incorporated in broader health care program? Intended for a particular patient population? Goals of the intervention, e.g., being more cost-effective to other interventions, replace or complement other solutions? (Note: Details about the intervention are provided in “Methods” under 5)

	1	2	3	4	5	
subitem not at all important	○	○	○	○	◉	essential

Clear selection

Your response is too large. Try shortening some answers.

Does your paper address subitem 2a-i? *

Copy and paste relevant sections from the manuscript (include quotes in quotation marks "like this" to indicate direct quotes from your manuscript), or elaborate on this item by providing additional information not in the ms, or briefly explain why the item is not applicable/relevant for your study

Yes. "Emerging adulthood often marks the onset of body image concerns [36]. Presently, most intervention programs are designed for female participants and are conducted in Western populations. The Body Project was only recently modified to cater to male participants and mixed-sex groups [42-44]. However, these programs are conducted face-to-face and not on mobile platforms. Evidence is emerging that mobile apps can provide convenient, effective, and cost-friendly mental health interventions [45]. Therefore, this study evaluated the effectiveness of a self-guided mHealth body image app for both female and male participants. The app adopted both cognitive dissonance and self-compassion approaches, covering the following three topics: media literacy, appearance comparisons, and self-compassion. These topics were selected because of the robust empirical evidence that has been found for their role as risk and protective factors of body image concerns. The content was adapted from existing evidence-based interventions for body image, eating disorders, and self-compassion [46-50]. We predicted that the intervention would lead to significant improvements on measures of body image and self-compassion after the intervention and 4-week follow-up, compared with an active waitlist control group."

2a-ii) Scientific background, rationale: What is known about the (type of) system

Scientific background, rationale: What is known about the (type of) system that is the object of the study (be sure to discuss the use of similar systems for other conditions/diagnoses, if appropiate), motivation for the study, i.e. what are the reasons for and what is the context for this specific study, from which stakeholder viewpoint is the study performed, potential impact of findings [2]. Briefly justify the choice of the comparator.

	1	2	3	4	5	
subitem not at all important	○	○	○	○	◉	essential

Clear selection

Your response is too large. Try shortening some answers.

Does your paper address subitem 2a-ii? *

Copy and paste relevant sections from the manuscript (include quotes in quotation marks "like this" to indicate direct quotes from your manuscript), or elaborate on this item by providing additional information not in the ms, or briefly explain why the item is not applicable/relevant for your study

Yes
"Background
Body image problems are highly prevalent among adolescents and young adults and have been frequently implicated in the development and maintenance of problematic eating behaviors [1,2] and body dysmorphic disorder [3,4]. Young adults may be particularly vulnerable to developing body image problems because of particular risk factors associated with this life period [5,6]. As young people transition through this developmental period, their bodies change in height, weight, and proportion while being exposed to social pressures associated with physical appearances [7]. Sociocultural factors play an important role in the development of body image concerns. According to the tripartite influence model, media, peers, and parents are the main sources of social influence on an individual's body image [8]. These influences largely take place through appearance-ideal internalization and appearance comparisons [9]. Research has shown that women tend to desire the thin-ideal, whereas men desire muscularity and weight [10]. Appearance-ideal internalization was found to mediate the relationship between sociocultural influences and body dissatisfaction [11,12], with greater internalization exacerbating body dissatisfaction [9]. This suggests that reducing the internalization of appearance ideals likely decreases body dissatisfaction.

Individuals tend to engage in upward appearance comparison, whereby they compare their appearance to others whom they perceive to be more attractive [13]. Consequently, they experience lower body esteem and higher body dissatisfaction, which perpetuate further appearance comparison [9,14].

Exposure to media contributes to appearance-ideal internalization and appearance comparison. Media and social networking platforms are filled with ideal-looking images of the self and others which are often skewed representations of reality [15,16]. Through social learning, individuals tend to normalize such content and internalize them as reality [15,17]. Moreover, individuals with vulnerability factors such as preexisting body image concerns, low self-esteem, depression, perfectionism, or overvalued appearance ideals are more likely to engage in appearance comparison to seek assurance and validation [18]. Altogether, appearance comparison on media platforms contributes to the development and maintenance of body dissatisfaction [9], highlighting the importance of addressing media literacy in reducing body image concerns.

Among peers, appearance-focused comparison and appearance-related conversations and activities may also increase body dissatisfaction as they increase individuals' awareness of their bodies, strengthen the internalization of appearance ideals, and negatively alter personal attitudes and beliefs in relation to beauty standards [19].

Apart from sociocultural influences, ruminative cognitive styles have been associated with greater body dissatisfaction [20]. Rumination is a response style to distress wherein individuals focus on repetitive thoughts and feelings about the distress [21]. As such, distress arising from negative body image may elicit rumination about one's appearance, which in turn contributes to body dissatisfaction [22].

Your response is too large. Try shortening some answers.

[illegible]
bodies through attending to the body's needs, protecting ourselves against unrealistic body

[illegible] ideals, having broader conceptualizations of beauty, and filtering information in a body-protective manner [23,24]. In qualitative studies, participants with positive body image actively rejected unrealistic media images to protect their body image [25,26]. Growing literature highlights that self-compassion contributes to a positive body image [27]. Self-compassion has been found to buffer the impact of media pressure on thin-ideal internalization in women [24], and to reduce body image distress and body dissatisfaction, reduce rumination, and increase body appreciation [24,25,46]. Homan and Tylka [30] highlighted that women who were high in self-compassion maintained high levels of body appreciation in the face of body-related comparisons. Thus, enhancing individuals' self-compassion may reduce the effects of negative body image and promote positive body image.

Intervention programs targeting body image risk and protective factors have been developed in the last 2 decades. Psychoeducational and cognitive behavioral programs have been effective in improving body image concerns, reducing disordered eating behaviors and attitudes, thin-ideal internalization, and dieting in adolescents and young adults [31,32]. Dissonance-based interventions are also increasingly adopted to address health and social behaviors [33]. For example, the Body Project adopted dissonance-based approaches in a group setting by having participants voluntarily critique and take a counterattitudinal stance against the thin-ideal in verbal, written, and behavioral activities [34]. It was theorized that the discrepancy generated between participants' personal beliefs (eg, thinner is better) and the counterattitudinal arguments made against pursuing thinness would elicit discomfort, and the discomfort would be alleviated by adjusting their personal beliefs to be more in line with the anti–thin-ideal statements [34]. Efficacy trials of the Body Project showed reduced eating disorder risk factors (eg, thin-ideal internalization and body dissatisfaction) and fewer eating disorder symptoms in female adolescents and young adults with body image concerns compared with assessment-only control conditions or alternative interventions, with numerous effects sustained up to 3-year follow-ups [35,36]. The Body Project M designed for male participants found that cognitive dissonance approach improved outcomes related to male participants' dissatisfaction with body fat and muscularity, body appreciation, muscularity-enhancing behaviors, appearance comparison, and internalization after the intervention, with all outcomes except dissatisfaction with muscularity and internalization being sustained at 3-month follow-up [33]. Encouraging findings were also found for the Body Project: More Than Muscles, wherein significant reductions were observed for several eating disorder risk factors and muscularity and body fat dissatisfaction in male participants, with some outcomes maintained at the 4-week follow-up [34]. A further extension of the Body Project, the Body Project 4 All, evaluated the effectiveness of a mixed-sex program which found gains to be sustained over a 6-month follow-up [35]. Meta-analyses confirmed the effectiveness of dissonance-based programs [36,37]. Altogether, these studies suggest that dissonance-based interventions are promising in improving body image concerns in male and female participants.

The direct challenging of the thin-ideal within dissonance-based interventions differs somewhat from a self-compassion approach, which aims to promote greater awareness of adverse outcomes created by the thin-ideal. In response to this awareness, self-compassion interventions engender a mindset that promotes self-kindness and connection with others in the face of body image concerns. In other words, self-compassion approaches seek to alter the way in which individuals cope with the distress associated with negative body image [28,29] rather than changing body image itself.

Self-compassion interventions are gaining empirical support in alleviating body image concerns. Self-compassion meditation and single-session self-compassion writing tasks [illegible]

Your response is too large. Try shortening some answers.

[illegible] comparable with and more effective than waitlist control and suggested that integrating

both self-compassion and dissonance-based approaches in interventions for body image may increase the acceptability of the interventions and reap more beneficial outcomes.

Self-guided, mobile-based body image programs have been developed and evaluated in light of technological advancements [39-41]. The 7-day mobile app study by Kosinski [40] led to a decrease in participants' body dissatisfaction, drive for thinness, and increase in self-esteem. Cerea et al [39] adopted a cognitive behavioral training approach with short, daily, cognitive training exercises for 16 days and found that it reduced body dissatisfaction in female university students. Finally, BodiMojo, a 6-week program, which involves sending daily intervention messages on body image and self-compassion–related content, increased appearance esteem and self-compassion in adolescents [41]. In a sample of high school and college students, BodiMojo improved participants' body image and self-compassion [41]."

2b) In INTRODUCTION: Specific objectives or hypotheses

Does your paper address CONSORT subitem 2b? *

Copy and paste relevant sections from the manuscript (include quotes in quotation marks "like this" to indicate direct quotes from your manuscript), or elaborate on this item by providing additional information not in the ms, or briefly explain why the item is not applicable/relevant for your study

Yes. "Therefore, this study evaluated the effectiveness of a self-guided mHealth body image app for both female and male participants." and "We predicted that the intervention would lead to significant improvements on measures of body image and self-compassion after the intervention and 4-week follow-up, compared with an active waitlist control group."

METHODS

3a) Description of trial design (such as parallel, factorial) including allocation ratio

Your response is too large. Try shortening some answers.

Does your paper address CONSORT subitem 3a? *

Copy and paste relevant sections from the manuscript (include quotes in quotation marks "like this" to indicate direct quotes from your manuscript), or elaborate on this item by providing additional information not in the ms, or briefly explain why the item is not applicable/relevant for your study

Yes. "Thereafter, participants were randomized to 1 of 2 conditions, intervention or active waitlist control, using simple randomization procedures. In this study, blinding of participants was marginally feasible as the content of the intervention programs that the participants engaged in were different in nature. However, participants were not outwardly informed of the real function of each intervention condition or of the real nature of the study being to evaluate the effectiveness of the body image program. The title of the study made known to participants was kept general (The effectiveness of a self-guided mobile phone application in improving the way we see ourselves and our bodies) to reduce the demand characteristics of the participants."

3b) Important changes to methods after trial commencement (such as eligibility criteria), with reasons

Does your paper address CONSORT subitem 3b? *

Copy and paste relevant sections from the manuscript (include quotes in quotation marks "like this" to indicate direct quotes from your manuscript), or elaborate on this item by providing additional information not in the ms, or briefly explain why the item is not applicable/relevant for your study

No important changes to methods after trial commencement

3b-i) Bug fixes, Downtimes, Content Changes

Bug fixes, Downtimes, Content Changes: ehealth systems are often dynamic systems. A description of changes to methods therefore also includes important changes made on the intervention or comparator during the trial (e.g., major bug fixes or changes in the functionality or content) (5-iii) and other “unexpected events” that may have influenced study design such as staff changes, system failures/downtimes, etc. [2].

	1	2	3	4	5	
subitem not at all important	○	○	○	○	◉	essential

Clear selection

Your response is too large. Try shortening some answers.

Does your paper address subitem 3b-i?

Copy and paste relevant sections from the manuscript (include quotes in quotation marks "like this" to indicate direct quotes from your manuscript), or elaborate on this item by providing additional information not in the ms, or briefly explain why the item is not applicable/relevant for your study

No important changes made on the intervention or comparator during the trial

4a) Eligibility criteria for participants

Does your paper address CONSORT subitem 4a? *

Copy and paste relevant sections from the manuscript (include quotes in quotation marks "like this" to indicate direct quotes from your manuscript), or elaborate on this item by providing additional information not in the ms, or briefly explain why the item is not applicable/relevant for your study

Yes. "The sample consisted of 310 female (age: mean 21.12, SD 2.07 years) and male (age: mean 22.68, SD 2.10 years) adults aged between 18 to 30 years, recruited from the department of psychology's research participant pool and the research recruitment platform of the National University of Singapore"

4a-i) Computer / Internet literacy

Computer / Internet literacy is often an implicit "de facto" eligibility criterion - this should be explicitly clarified.

	1	2	3	4	5	
subitem not at all important	○	○	◉	○	○	essential

Clear selection

Your response is too large. Try shortening some answers.

Does your paper address subitem 4a-i?

Copy and paste relevant sections from the manuscript (include quotes in quotation marks "like this" to indicate direct quotes from your manuscript), or elaborate on this item by providing additional information not in the ms, or briefly explain why the item is not applicable/relevant for your study

Participants in our study are required to register for the study via an web-based link in order to take part in the study.

4a-ii) Open vs. closed, web-based vs. face-to-face assessments:

Open vs. closed, web-based vs. face-to-face assessments: Mention how participants were recruited (online vs. offline), e.g., from an open access website or from a clinic, and clarify if this was a purely web-based trial, or there were face-to-face components (as part of the intervention or for assessment), i.e., to what degree got the study team to know the participant. In online-only trials, clarify if participants were quasi-anonymous and whether having multiple identities was possible or whether technical or logistical measures (e.g., cookies, email confirmation, phone calls) were used to detect/prevent these.

	1	2	3	4	5	
subitem not at all important	○	○	○	○	◉	essential

Clear selection

Does your paper address subitem 4a-ii? *

Copy and paste relevant sections from the manuscript (include quotes in quotation marks "like this" to indicate direct quotes from your manuscript), or elaborate on this item by providing additional information not in the ms, or briefly explain why the item is not applicable/relevant for your study

Yes. "The sample consisted of 310 female (age: mean 21.12, SD 2.07 years) and male (age: mean 22.68, SD 2.10 years) adults aged between 18 to 30 years, recruited from the department of psychology's research participant pool and the research recruitment platform of the National University of Singapore." and "Data collection took place in Singapore in an entirely web-based setting."

Your response is too large. Try shortening some answers.

4a-iii) Information giving during recruitment

Information given during recruitment. Specify how participants were briefed for recruitment and in the informed consent procedures (e.g., publish the informed consent documentation as appendix, see also item X26), as this information may have an effect on user self-selection, user expectation and may also bias results.

	1	2	3	4	5	
subitem not at all important	○	○	○	○	◉	essential

Clear selection

Does your paper address subitem 4a-iii?

Copy and paste relevant sections from the manuscript (include quotes in quotation marks "like this" to indicate direct quotes from your manuscript), or elaborate on this item by providing additional information not in the ms, or briefly explain why the item is not applicable/relevant for your study

Yes.
"Procedure and Participant Flow
Participants first read the Participation Information Sheet on Qualtrics. After providing informed consent, participants completed measures on body image, body image risk factors, and self-compassion to obtain baseline ratings. Thereafter, participants were randomized to 1 of 2 conditions, intervention or active waitlist control, using simple randomization procedures. In this study, blinding of participants was marginally feasible as the content of the intervention programs that the participants engaged in were different in nature. However, participants were not outwardly informed of the real function of each intervention condition or of the real nature of the study being to evaluate the effectiveness of the body image program. The title of the study made known to participants was kept general (The effectiveness of a self-guided mobile phone application in improving the way we see ourselves and our bodies) to reduce the demand characteristics of the participants.

Next, the participants downloaded the mobile app and were guided on how to navigate the app. Participants in the intervention group underwent 9 days of body image training, while participants in the active waitlist control group underwent 9 days of the cooperation learning program. The anticipated time participants spent on each program was comparable (<5 minutes per day)."

4b) Settings and locations where the data were collected

Your response is too large. Try shortening some answers.

Does your paper address CONSORT subitem 4b? *

Copy and paste relevant sections from the manuscript (include quotes in quotation marks "like this" to indicate direct quotes from your manuscript), or elaborate on this item by providing additional information not in the ms, or briefly explain why the item is not applicable/relevant for your study

Yes. "Data collection took place in Singapore in an entirely web-based setting."

4b-i) Report if outcomes were (self-)assessed through online questionnaires

Clearly report if outcomes were (self-)assessed through online questionnaires (as common in web-based trials) or otherwise.

	1	2	3	4	5	
subitem not at all important	○	○	○	○	◉	essential

Clear selection

Does your paper address subitem 4b-i? *

Copy and paste relevant sections from the manuscript (include quotes in quotation marks "like this" to indicate direct quotes from your manuscript), or elaborate on this item by providing additional information not in the ms, or briefly explain why the item is not applicable/relevant for your study

Yes. "After providing informed consent, participants completed measures on body image, body image risk factors, and self-compassion to obtain baseline ratings." and "Participants filled out the same questionnaires upon program completion (postintervention measure) and after 4 weeks (follow-up measure). The feedback questionnaire was administered only after the intervention."

Your response is too large. Try shortening some answers.

4b-ii) Report how institutional affiliations are displayed

Report how institutional affiliations are displayed to potential participants [on ehealth media], as affiliations with prestigious hospitals or universities may affect volunteer rates, use, and reactions with regards to an intervention.(Not a required item – describe only if this may bias results)

	1	2	3	4	5	
subitem not at all important	◉	○	○	○	○	essential

Clear selection

Does your paper address subitem 4b-ii?

Copy and paste relevant sections from the manuscript (include quotes in quotation marks "like this" to indicate direct quotes from your manuscript), or elaborate on this item by providing additional information not in the ms, or briefly explain why the item is not applicable/relevant for your study

Not applicable to our study. There are no institutional affiliations relevant in our study.

5) The interventions for each group with sufficient details to allow replication, including how and when they were actually administered

5-i) Mention names, credential, affiliations of the developers, sponsors, and owners

Mention names, credential, affiliations of the developers, sponsors, and owners [6] (if authors/evaluators are owners or developer of the software, this needs to be declared in a "Conflict of interest" section or mentioned elsewhere in the manuscript).

	1	2	3	4	5	
subitem not at all important	○	○	○	○	◉	essential

Clear selection

Your response is too large. Try shortening some answers.

Does your paper address subitem 5-i?

Copy and paste relevant sections from the manuscript (include quotes in quotation marks "like this" to indicate direct quotes from your manuscript), or elaborate on this item by providing additional information not in the ms, or briefly explain why the item is not applicable/relevant for your study

Yes. "The study was partly funded by Intellect Pte Ltd. The study design, data management, interpretation, analysis, and reporting and the decision to publish the study are entirely independent of Intellect Pte Ltd." and "OS had a research collaboration with Intellect Pte Ltd at the time of the data collection and has since joined Intellect Pte Ltd as their clinical director."

5-ii) Describe the history/development process

Describe the history/development process of the application and previous formative evaluations (e.g., focus groups, usability testing), as these will have an impact on adoption/use rates and help with interpreting results.

	1	2	3	4	5	
subitem not at all important	○	○	○	○	◉	essential

Clear selection

Your response is too large. Try shortening some answers.

Does your paper address subitem 5-ii?

Copy and paste relevant sections from the manuscript (include quotes in quotation marks "like this" to indicate direct quotes from your manuscript), or elaborate on this item by providing additional information not in the ms, or briefly explain why the item is not applicable/relevant for your study

Yes. "The app adopted both cognitive dissonance and self-compassion approaches, covering the following three topics: media literacy, appearance comparisons, and self-compassion. These topics were selected because of the robust empirical evidence that has been found for their role as risk and protective factors of body image concerns. The content was adapted from existing evidence-based interventions for body image, eating disorders, and self-compassion";

"Body Image Program
This 9-day program adopted cognitive dissonance and self-compassion approaches designed around the following three topics: media literacy, appearance comparison, and self-compassion. At the start of each 3-day period, the participants underwent a 5-minute content learning and dissonance-based or self-compassion activity related to the topic (Textbox 1). The dissonance-based activity involved participants challenging sociocultural influences regarding media messages, appearance ideals, and appearance comparison. Participants typed their answers to questions which guided them in challenging sociocultural ideals. Self-compassion interventions involved psychoeducation and experiential activities. Participants were also given a cognitive or behavioral task which encouraged noticing and challenging sociocultural influences in their daily lives, or practicing self-compassion. Daily body image and self-compassion–focused messages were sent through the app to participants thrice a day, messages modeled after the BodiMojo mobile app (Table 1) [41]. These intervention messages included psychoeducation, affirmations, behavioral tips, short activities, and quizzes to reinforce participants' learning (Figure 1)."

"Cooperation Learning Program
Participants in the active waitlist control group engaged in a self-guided learning program on cooperation. The active waitlist control group was chosen instead of a waitlist control as it serves as an attention control to create similar experiences for participants in both groups to control for nonspecific factors that may influence the study outcomes [54]. This 9-day learning program develops participants' skills to improve group morale and relationships. It consists of content learning once a day and activities such as quizzes, and the number and length of activities was matched to the body image app."

Your response is too large. Try shortening some answers.

5-iii) Revisions and updating

Revisions and updating. Clearly mention the date and/or version number of the application/intervention (and comparator, if applicable) evaluated, or describe whether the intervention underwent major changes during the evaluation process, or whether the development and/or content was “frozen” during the trial. Describe dynamic components such as news feeds or changing content which may have an impact on the replicability of the intervention (for unexpected events see item 3b).

	1	2	3	4	5	
subitem not at all important	○	○	◉	○	○	essential

Clear selection

Does your paper address subitem 5-iii?

Copy and paste relevant sections from the manuscript (include quotes in quotation marks "like this" to indicate direct quotes from your manuscript), or elaborate on this item by providing additional information not in the ms, or briefly explain why the item is not applicable/relevant for your study

Not appplicable for our study- there is only one version of the program to date.

5-iv) Quality assurance methods

Provide information on quality assurance methods to ensure accuracy and quality of information provided [1], if applicable.

	1	2	3	4	5	
subitem not at all important	○	○	○	○	◉	essential

Clear selection

Your response is too large. Try shortening some answers.

Does your paper address subitem 5-iv?

Copy and paste relevant sections from the manuscript (include quotes in quotation marks "like this" to indicate direct quotes from your manuscript), or elaborate on this item by providing additional information not in the ms, or briefly explain why the item is not applicable/relevant for your study

Yes. "Most outcome variables met the assumption tests for ANCOVA. The homogeneity of variance assumption was violated for a small number of outcome measures. However, because of the robustness of ANCOVA when sample sizes in each group are relatively equal [65], analysis using ANCOVA proceeded. The assumption of independence between independent variable and covariate was met for all outcome measures (P>.20), except BIQ for male participants. The assumption of homogeneity of regression slopes were violated in 2 variables for female participants (postintervention BIQ; postintervention SATAQ-4R Internalisation: Thin/Low Body Fat). For these variables, 1-way ANOVA was conducted using differences in scores between baseline and after the intervention and baseline and follow-up, respectively." and "The coauthor (OS) also provided supervision of the overall study"

5-v) Ensure replicability by publishing the source code, and/or providing screenshots/screen-capture video, and/or providing flowcharts of the algorithms used

Ensure replicability by publishing the source code, and/or providing screenshots/screen-capture video, and/or providing flowcharts of the algorithms used. Replicability (i.e., other researchers should in principle be able to replicate the study) is a hallmark of scientific reporting.

	1	2	3	4	5	
subitem not at all important	○	○	◉	○	○	essential

Clear selection

Does your paper address subitem 5-v?

Copy and paste relevant sections from the manuscript (include quotes in quotation marks "like this" to indicate direct quotes from your manuscript), or elaborate on this item by providing additional information not in the ms, or briefly explain why the item is not applicable/relevant for your study

Yes. Screenshots and flowcharts have been uploaded through the submission system.

Your response is too large. Try shortening some answers.

5-vi) Digital preservation

Digital preservation: Provide the URL of the application, but as the intervention is likely to change or disappear over the course of the years; also make sure the intervention is archived (Internet Archive, webcitation.org, and/or publishing the source code or screenshots/videos alongside the article). As pages behind login screens cannot be archived, consider creating demo pages which are accessible without login.

	1	2	3	4	5	
subitem not at all important	○	○	◉	○	○	essential

Clear selection

Does your paper address subitem 5-vi?

Copy and paste relevant sections from the manuscript (include quotes in quotation marks "like this" to indicate direct quotes from your manuscript), or elaborate on this item by providing additional information not in the ms, or briefly explain why the item is not applicable/relevant for your study

Yes. Screenshots of the app program are provided. The app program is currently not readily available as a URL.

5-vii) Access

Access: Describe how participants accessed the application, in what setting/context, if they had to pay (or were paid) or not, whether they had to be a member of specific group. If known, describe how participants obtained “access to the platform and Internet” [1]. To ensure access for editors/reviewers/readers, consider to provide a “backdoor” login account or demo mode for reviewers/readers to explore the application (also important for archiving purposes, see vi).

	1	2	3	4	5	
subitem not at all important	○	○	◉	○	○	essential

Clear selection

Your response is too large. Try shortening some answers.

Does your paper address subitem 5-vii? *

Copy and paste relevant sections from the manuscript (include quotes in quotation marks "like this" to indicate direct quotes from your manuscript), or elaborate on this item by providing additional information not in the ms, or briefly explain why the item is not applicable/relevant for your study

Yes. "Participants first read the Participation Information Sheet on Qualtrics. After providing informed consent, participants completed measures on body image, body image risk factors, and self-compassion to obtain baseline ratings. Thereafter, participants were randomized to 1 of 2 conditions, intervention or active waitlist control, using simple randomization procedures.", "Next, the participants downloaded the mobile app and were guided on how to navigate the app. Participants in the intervention group underwent 9 days of body image training, while participants in the active waitlist control group underwent 9 days of the cooperation learning program", "

5-viii) Mode of delivery, features/functionalities/components of the intervention and comparator, and the theoretical framework

Describe mode of delivery, features/functionalities/components of the intervention and comparator, and the theoretical framework [6] used to design them (instructional strategy [1], behaviour change techniques, persuasive features, etc., see e.g., [7, 8] for terminology). This includes an in-depth description of the content (including where it is coming from and who developed it) [1]," whether [and how] it is tailored to individual circumstances and allows users to track their progress and receive feedback" [6]. This also includes a description of communication delivery channels and – if computer-mediated communication is a component – whether communication was synchronous or asynchronous [6]. It also includes information on presentation strategies [1], including page design principles, average amount of text on pages, presence of hyperlinks to other resources, etc. [1].

	1	2	3	4	5	
subitem not at all important	○	○	○	○	◉	essential

Clear selection

Your response is too large. Try shortening some answers.

Does your paper address subitem 5-viii? *

Copy and paste relevant sections from the manuscript (include quotes in quotation marks "like this" to indicate direct quotes from your manuscript), or elaborate on this item by providing additional information not in the ms, or briefly explain why the item is not applicable/relevant for your study

Yes. "The app adopted both cognitive dissonance and self-compassion approaches, covering the following three topics: media literacy, appearance comparisons, and self-compassion. These topics were selected because of the robust empirical evidence that has been found for their role as risk and protective factors of body image concerns. The content was adapted from existing evidence-based interventions for body image, eating disorders, and self-compassion";

"Body Image Program
This 9-day program adopted cognitive dissonance and self-compassion approaches designed around the following three topics: media literacy, appearance comparison, and self-compassion. At the start of each 3-day period, the participants underwent a 5-minute content learning and dissonance-based or self-compassion activity related to the topic (Textbox 1). The dissonance-based activity involved participants challenging sociocultural influences regarding media messages, appearance ideals, and appearance comparison. Participants typed their answers to questions which guided them in challenging sociocultural ideals. Self-compassion interventions involved psychoeducation and experiential activities. Participants were also given a cognitive or behavioral task which encouraged noticing and challenging sociocultural influences in their daily lives, or practicing self-compassion. Daily body image and self-compassion–focused messages were sent through the app to participants thrice a day, messages modeled after the BodiMojo mobile app (Table 1) [41]. These intervention messages included psychoeducation, affirmations, behavioral tips, short activities, and quizzes to reinforce participants' learning (Figure 1)."

"Cooperation Learning Program
Participants in the active waitlist control group engaged in a self-guided learning program on cooperation. The active waitlist control group was chosen instead of a waitlist control as it serves as an attention control to create similar experiences for participants in both groups to control for nonspecific factors that may influence the study outcomes [54]. This 9-day learning program develops participants' skills to improve group morale and relationships. It consists of content learning once a day and activities such as quizzes, and the number and length of activities was matched to the body image app."

Your response is too large. Try shortening some answers.

5-ix) Describe use parameters

Describe use parameters (e.g., intended “doses” and optimal timing for use). Clarify what instructions or recommendations were given to the user, e.g., regarding timing, frequency, heaviness of use, if any, or was the intervention used ad libitum.

	1	2	3	4	5	
subitem not at all important	○	○	◉	○	○	essential

Clear selection

Does your paper address subitem 5-ix?

Copy and paste relevant sections from the manuscript (include quotes in quotation marks "like this" to indicate direct quotes from your manuscript), or elaborate on this item by providing additional information not in the ms, or briefly explain why the item is not applicable/relevant for your study

Yes. "The anticipated time participants spent on each program was comparable (<5 minutes per day)."

"Body Image Program
....At the start of each 3-day period, the participants underwent a 5-minute content learning and dissonance-based or self-compassion activity related to the topic (Textbox 1). The dissonance-based activity involved participants challenging sociocultural influences regarding media messages, appearance ideals, and appearance comparison. Participants typed their answers to questions which guided them in challenging sociocultural ideals. Self-compassion interventions involved psychoeducation and experiential activities. Participants were also given a cognitive or behavioral task which encouraged noticing and challenging sociocultural influences in their daily lives, or practicing self-compassion. Daily body image and self-compassion–focused messages were sent through the app to participants thrice a day, messages modeled after the BodiMojo mobile app (Table 1) [41]. These intervention messages included psychoeducation, affirmations, behavioral tips, short activities, and quizzes to reinforce participants’ learning (Figure 1)."

"Cooperation Learning Program
....This 9-day learning program develops participants’ skills to improve group morale and relationships. It consists of content learning once a day and activities such as quizzes, and the number and length of activities was matched to the body image app."

Your response is too large. Try shortening some answers.

5-x) Clarify the level of human involvement

Clarify the level of human involvement (care providers or health professionals, also technical assistance) in the e-intervention or as co-intervention (detail number and expertise of professionals involved, if any, as well as “type of assistance offered, the timing and frequency of the support, how it is initiated, and the medium by which the assistance is delivered”. It may be necessary to distinguish between the level of human involvement required for the trial, and the level of human involvement required for a routine application outside of a RCT setting (discuss under item 21 – generalizability).

	1	2	3	4	5	
subitem not at all important	○	○	◉	○	○	essential

Clear selection

Does your paper address subitem 5-x?

Copy and paste relevant sections from the manuscript (include quotes in quotation marks "like this" to indicate direct quotes from your manuscript), or elaborate on this item by providing additional information not in the ms, or briefly explain why the item is not applicable/relevant for your study

Yes. "Data collection took place in Singapore in an entirely web-based setting."

5-xi) Report any prompts/reminders used

Report any prompts/reminders used: Clarify if there were prompts (letters, emails, phone calls, SMS) to use the application, what triggered them, frequency etc. It may be necessary to distinguish between the level of prompts/reminders required for the trial, and the level of prompts/reminders for a routine application outside of a RCT setting (discuss under item 21 – generalizability).

	1	2	3	4	5	
subitem not at all important	○	○	○	○	◉	essential

Clear selection

Your response is too large. Try shortening some answers.

Does your paper address subitem 5-xi? *

Copy and paste relevant sections from the manuscript (include quotes in quotation marks "like this" to indicate direct quotes from your manuscript), or elaborate on this item by providing additional information not in the ms, or briefly explain why the item is not applicable/relevant for your study

Yes. "Daily body image and self-compassion–focused messages were sent through the app to participants thrice a day, messages modeled after the BodiMojo mobile app (Table 1) [41]. These intervention messages included psychoeducation, affirmations, behavioral tips, short activities, and quizzes to reinforce participants' learning (Figure 1)."

5-xii) Describe any co-interventions (incl. training/support)

Describe any co-interventions (incl. training/support): Clearly state any interventions that are provided in addition to the targeted eHealth intervention, as ehealth intervention may not be designed as stand-alone intervention. This includes training sessions and support [1]. It may be necessary to distinguish between the level of training required for the trial, and the level of training for a routine application outside of a RCT setting (discuss under item 21 – generalizability.

	1	2	3	4	5	
subitem not at all important	○	○	●	○	○	essential

Clear selection

Does your paper address subitem 5-xii? *

Copy and paste relevant sections from the manuscript (include quotes in quotation marks "like this" to indicate direct quotes from your manuscript), or elaborate on this item by providing additional information not in the ms, or briefly explain why the item is not applicable/relevant for your study

Not applicable. No other co-interventions were required for our study.

6a) Completely defined pre-specified primary and secondary outcome measures, including how and when they were assessed

Your response is too large. Try shortening some answers.

Does your paper address CONSORT subitem 6a? *

Copy and paste relevant sections from the manuscript (include quotes in quotation marks "like this" to indicate direct quotes from your manuscript), or elaborate on this item by providing additional information not in the ms, or briefly explain why the item is not applicable/relevant for your study

Yes. "Outcome Measures
Overview
Body Image Ideals Questionnaire (BIQ [56]) is a 22-item scale that assesses body image satisfaction-dissatisfaction by measuring the degree of congruence or discrepancy in one's perceived and idealized physical attributes. On a scale ranging from 0 (exactly as I am) to 3 (very unlike me), participants rated the degree to which they resembled their physical ideal on 11 physical attributes. Next, participants rated the importance that they assigned to attaining their ideal on each physical attribute. The cross-products of the discrepancy and importance ratings for each physical attribute were obtained and a composite BIQ score was computed. Higher scores indicated greater disparity between one's perceived and ideal physical attributes, suggesting higher levels of body dissatisfaction. The BIQ showed good internal consistency, with a Cronbach α of .81 for male participants and .76 for female participants.

The Body Appreciation Scale-2 (24) is a 10-item scale that assesses individuals' positive attitudes toward their bodies. The items are scored on a scale from 1 (never) to 5 (always). Scores on all items are averaged with higher scores indicating greater body appreciation. The Body Appreciation Scale-2 has excellent internal consistency, with a Cronbach α of .96 for male participants and .97 for female participants.

The Sociocultural Attitudes Toward Appearance Questionnaire-4 Revised [57] measures internalization ideals and appearance-related sociocultural pressures. The 7 subscales consist of 31 items for female participants and 28 items for male participants on a scale ranging from 1 (definitely disagree) to 5 (definitely agree). Higher scores on each subscale indicate higher levels of internalization and sociocultural pressure. In this study, the subscales of Internalisation: Thin/Low Body Fat (for female participants), Internalisation: Muscularity (for male participants), and Pressures: Peers and Media were used. The internal consistencies of the subscales are good, with Cronbach α of ≥.82 in a sample of university female participants and Cronbach α of ≥.75 in a sample of university male participants.

Self-Compassion Scale-Short Form [58] is a 12-item scale that measures self-compassion on 6 subscales. Each item is scored from 1 (almost never) to 5 (almost always). A total self-compassion score is the mean of all 6 subscales, with higher scores indicating higher levels of self-compassion. The internal consistency of the scale is excellent, with a Cronbach α of .86.

App Engagement
App Engagement Scale [59] is a 7-item scale that measures participants' engagement on the phone app with scores ranging from 1 (definitely disagree) to 5 (definitely agree). A total score is derived by adding the scores from each item. Internal reliability of the scale is good, with a Cronbach α of .84."

Your response is too large. Try shortening some answers.

6a-i) Online questionnaires: describe if they were validated for online use and apply CHERRIES items to describe how the questionnaires were designed/deployed

If outcomes were obtained through online questionnaires, describe if they were validated for online use and apply CHERRIES items to describe how the questionnaires were designed/deployed [9].

	1	2	3	4	5	
subitem not at all important	○	○	◉	○	○	essential

Clear selection

Does your paper address subitem 6a-i?

Copy and paste relevant sections from manuscript text

The online questionnaires are self-report items that has been used by other web-based studies. CHERRIES items were not applied for our study.

6a-ii) Describe whether and how "use" (including intensity of use/dosage) was defined/measured/monitored

Describe whether and how "use" (including intensity of use/dosage) was defined/measured/monitored (logins, logfile analysis, etc.). Use/adoption metrics are important process outcomes that should be reported in any ehealth trial.

	1	2	3	4	5	
subitem not at all important	○	○	◉	○	○	essential

Clear selection

Does your paper address subitem 6a-ii?

Copy and paste relevant sections from manuscript text

Our paper did not address the item explicitly. However, back-end checks were conducted with The Intellect Company to ensure that participants completed all activities in the program before questionnaires were administered after intervention and before reimbursement.

Your response is too large. Try shortening some answers.

6a-iii) Describe whether, how, and when qualitative feedback from participants was obtained

Describe whether, how, and when qualitative feedback from participants was obtained (e.g., through emails, feedback forms, interviews, focus groups).

	1	2	3	4	5	
subitem not at all important	○	○	◉	○	○	essential

Clear selection

Does your paper address subitem 6a-iii?

Copy and paste relevant sections from manuscript text

Our paper did not obtain qualitative feedback. However, quantitative feedback was obtained through the App Engagement Questionnaire.

"App Engagement
App Engagement Scale [59] is a 7-item scale that measures participants' engagement on the phone app with scores ranging from 1 (definitely disagree) to 5 (definitely agree). A total score is derived by adding the scores from each item. Internal reliability of the scale is good, with a Cronbach α of .84."

6b) Any changes to trial outcomes after the trial commenced, with reasons

Does your paper address CONSORT subitem 6b? *

Copy and paste relevant sections from the manuscript (include quotes in quotation marks "like this" to indicate direct quotes from your manuscript), or elaborate on this item by providing additional information not in the ms, or briefly explain why the item is not applicable/relevant for your study

Not applicable. No changes to trial outcomes after trial commenced.

7a) How sample size was determined

NPT: When applicable, details of whether and how the clustering by care provides or centers was addressed

Your response is too large. Try shortening some answers.

7a-i) Describe whether and how expected attrition was taken into account when calculating the sample size

Describe whether and how expected attrition was taken into account when calculating the sample size.

	1	2	3	4	5	
subitem not at all important	○	○	○	○	◉	essential

Clear selection

Does your paper address subitem 7a-i?

Copy and paste relevant sections from manuscript title (include quotes in quotation marks "like this" to indicate direct quotes from your manuscript), or elaborate on this item by providing additional information not in the ms, or briefly explain why the item is not applicable/relevant for your study

Yes. "A power analysis with G*power 3.1 [51] revealed a minimum number of 128 participants, using a moderate effect size as found in relevant mobile-based body image studies [41,52,53]. We aimed for a total of 308 participants to account for a potential attrition of 20% [41]."

7b) When applicable, explanation of any interim analyses and stopping guidelines

Does your paper address CONSORT subitem 7b? *

Copy and paste relevant sections from the manuscript (include quotes in quotation marks "like this" to indicate direct quotes from your manuscript), or elaborate on this item by providing additional information not in the ms, or briefly explain why the item is not applicable/relevant for your study

Not applicable. No interim analyses and stopping guidelines are required for our study.

8a) Method used to generate the random allocation sequence

NPT: When applicable, how care providers were allocated to each trial group

Your response is too large. Try shortening some answers.

Does your paper address CONSORT subitem 8a? *

Copy and paste relevant sections from the manuscript (include quotes in quotation marks "like this" to indicate direct quotes from your manuscript), or elaborate on this item by providing additional information not in the ms, or briefly explain why the item is not applicable/relevant for your study

Not applicable. Care providers were not required in our trial groups.

8b) Type of randomisation; details of any restriction (such as blocking and block size)

Does your paper address CONSORT subitem 8b? *

Copy and paste relevant sections from the manuscript (include quotes in quotation marks "like this" to indicate direct quotes from your manuscript), or elaborate on this item by providing additional information not in the ms, or briefly explain why the item is not applicable/relevant for your study

Yes. "Thereafter, participants were randomized to 1 of 2 conditions, intervention or active waitlist control, using simple randomization procedures."

9) Mechanism used to implement the random allocation sequence (such as sequentially numbered containers), describing any steps taken to conceal the sequence until interventions were assigned

Does your paper address CONSORT subitem 9? *

Copy and paste relevant sections from the manuscript (include quotes in quotation marks "like this" to indicate direct quotes from your manuscript), or elaborate on this item by providing additional information not in the ms, or briefly explain why the item is not applicable/relevant for your study

This was not explicitly written in the manuscript, but simple randomisation procedures were performed on the web-based sign up system-- Qualtrics platform.

10) Who generated the random allocation sequence, who enrolled participants, and who assigned participants to interventions

Your response is too large. Try shortening some answers.

Does your paper address CONSORT subitem 10? *

Copy and paste relevant sections from the manuscript (include quotes in quotation marks "like this" to indicate direct quotes from your manuscript), or elaborate on this item by providing additional information not in the ms, or briefly explain why the item is not applicable/relevant for your study

This was not explicitly written in the manuscript, but simple randomisation procedures were performed on the web-based sign up system-- Qualtrics platform.

11a) If done, who was blinded after assignment to interventions (for example, participants, care providers, those assessing outcomes) and how

NPT: Whether or not administering co-interventions were blinded to group assignment

11a-i) Specify who was blinded, and who wasn't

Specify who was blinded, and who wasn't. Usually, in web-based trials it is not possible to blind the participants [1, 3] (this should be clearly acknowledged), but it may be possible to blind outcome assessors, those doing data analysis or those administering co-interventions (if any).

	1	2	3	4	5	
subitem not at all important	○	○	◉	○	○	essential

Clear selection

Does your paper address subitem 11a-i? *

Copy and paste relevant sections from the manuscript (include quotes in quotation marks "like this" to indicate direct quotes from your manuscript), or elaborate on this item by providing additional information not in the ms, or briefly explain why the item is not applicable/relevant for your study

This was not explicitly indicated on the manuscript, but the authors were not blinded. It was addressed that "blinding of participants was marginally feasible as the content of the intervention programs that the participants engaged in were different in nature. However, participants were not outwardly informed of the real function of each intervention condition or of the real nature of the study being to evaluate the effectiveness of the body image program. The title of the study made known to participants was kept general (The effectiveness of a self-guided mobile phone application in improving the way we see ourselves and our bodies) to reduce the demand characteristics of the participants."

Your response is too large. Try shortening some answers.

11a-ii) Discuss e.g., whether participants knew which intervention was the "intervention of interest" and which one was the "comparator"

Informed consent procedures (4a-ii) can create biases and certain expectations - discuss e.g., whether participants knew which intervention was the "intervention of interest" and which one was the "comparator".

	1	2	3	4	5	
subitem not at all important	○	○	○	○	◉	essential

Clear selection

Does your paper address subitem 11a-ii?

Copy and paste relevant sections from the manuscript (include quotes in quotation marks "like this" to indicate direct quotes from your manuscript), or elaborate on this item by providing additional information not in the ms, or briefly explain why the item is not applicable/relevant for your study

Yes. "In this study, blinding of participants was marginally feasible as the content of the intervention programs that the participants engaged in were different in nature. However, participants were not outwardly informed of the real function of each intervention condition or of the real nature of the study being to evaluate the effectiveness of the body image program. The title of the study made known to participants was kept general (The effectiveness of a self-guided mobile phone application in improving the way we see ourselves and our bodies) to reduce the demand characteristics of the participants."

11b) If relevant, description of the similarity of interventions
(this item is usually not relevant for ehealth trials as it refers to similarity of a placebo or sham intervention to a active medication/intervention)

Your response is too large. Try shortening some answers.

Does your paper address CONSORT subitem 11b? *

Copy and paste relevant sections from the manuscript (include quotes in quotation marks "like this" to indicate direct quotes from your manuscript), or elaborate on this item by providing additional information not in the ms, or briefly explain why the item is not applicable/relevant for your study

Yes.
"It consists of content learning once a day and activities such as quizzes, and the number and length of activities was matched to the body image app." and "Participants in the intervention group underwent 9 days of body image training, while participants in the active waitlist control group underwent 9 days of the cooperation learning program. The anticipated time participants spent on each program was comparable (<5 minutes per day)."

12a) Statistical methods used to compare groups for primary and secondary outcomes

NPT: When applicable, details of whether and how the clustering by care providers or centers was addressed

Does your paper address CONSORT subitem 12a? *

Copy and paste relevant sections from the manuscript (include quotes in quotation marks "like this" to indicate direct quotes from your manuscript), or elaborate on this item by providing additional information not in the ms, or briefly explain why the item is not applicable/relevant for your study

Yes. Paragraphs on "Analytical approach" describes the statistical methods adopted.

12a-i) Imputation techniques to deal with attrition / missing values

Imputation techniques to deal with attrition / missing values: Not all participants will use the intervention/comparator as intended and attrition is typically high in ehealth trials. Specify how participants who did not use the application or dropped out from the trial were treated in the statistical analysis (a complete case analysis is strongly discouraged, and simple imputation techniques such as LOCF may also be problematic [4]).

	1	2	3	4	5	
subitem not at all important	○	○	○	○	◉	essential

Clear selection

Your response is too large. Try shortening some answers.

Does your paper address subitem 12a-i? *

Copy and paste relevant sections from the manuscript (include quotes in quotation marks "like this" to indicate direct quotes from your manuscript), or elaborate on this item by providing additional information not in the ms, or briefly explain why the item is not applicable/relevant for your study

Yes. "Intent-to-treat analyses were conducted to address loss of participant data because of participant withdrawal or technical difficulties, by carrying forward the participants' last reported score. "

12b) Methods for additional analyses, such as subgroup analyses and adjusted analyses

Does your paper address CONSORT subitem 12b? *

Copy and paste relevant sections from the manuscript (include quotes in quotation marks "like this" to indicate direct quotes from your manuscript), or elaborate on this item by providing additional information not in the ms, or briefly explain why the item is not applicable/relevant for your study

Not applicable. No additional analyses were conducted in our study.

X26) REB/IRB Approval and Ethical Considerations [recommended as subheading under "Methods"] (not a CONSORT item)

X26-i) Comment on ethics committee approval

	1	2	3	4	5	
subitem not at all important	○	○	○	○	◉	essential

Clear selection

Your response is too large. Try shortening some answers.

Does your paper address subitem X26-i?

Copy and paste relevant sections from the manuscript (include quotes in quotation marks "like this" to indicate direct quotes from your manuscript), or elaborate on this item by providing additional information not in the ms, or briefly explain why the item is not applicable/relevant for your study

Yes. "Ethics approval for this study was obtained from the National University of Singapore's (NUS) Institutional Review Board (NUS-IRB-2021-85), and it was preregistered with ClinicalTrials.gov (registration number: NCT04977973)."

x26-ii) Outline informed consent procedures

Outline informed consent procedures e.g., if consent was obtained offline or online (how? Checkbox, etc.?), and what information was provided (see 4a-ii). See [6] for some items to be included in informed consent documents.

	1	2	3	4	5	
subitem not at all important	○	○	○	○	●	essential

Clear selection

Does your paper address subitem X26-ii?

Copy and paste relevant sections from the manuscript (include quotes in quotation marks "like this" to indicate direct quotes from your manuscript), or elaborate on this item by providing additional information not in the ms, or briefly explain why the item is not applicable/relevant for your study

Yes. "Participants first read the Participation Information Sheet on Qualtrics. After providing informed consent, participants completed measures on body image, body image risk factors, and self-compassion to obtain baseline ratings."

X26-iii) Safety and security procedures

Safety and security procedures, incl. privacy considerations, and any steps taken to reduce the likelihood or detection of harm (e.g., education and training, availability of a hotline)

	1	2	3	4	5	
subitem not at all important	○	○	●	○	○	essential

Your response is too large. Try shortening some answers.

Does your paper address subitem X26-iii?

Copy and paste relevant sections from the manuscript (include quotes in quotation marks "like this" to indicate direct quotes from your manuscript), or elaborate on this item by providing additional information not in the ms, or briefly explain why the item is not applicable/relevant for your study

This was not explicitly written in our manuscript as there are no foreseeable discomforts and risks for participants.

RESULTS

13a) For each group, the numbers of participants who were randomly assigned, received intended treatment, and were analysed for the primary outcome

NPT: The number of care providers or centers performing the intervention in each group and the number of patients treated by each care provider in each center

Does your paper address CONSORT subitem 13a? *

Copy and paste relevant sections from the manuscript (include quotes in quotation marks "like this" to indicate direct quotes from your manuscript), or elaborate on this item by providing additional information not in the ms, or briefly explain why the item is not applicable/relevant for your study

Yes. These information are clearly indicated on the Consort diagram and tables to the submission.

13b) For each group, losses and exclusions after randomisation, together with reasons

Does your paper address CONSORT subitem 13b? (NOTE: Preferably, this is shown in a CONSORT flow diagram) *

Copy and paste relevant sections from the manuscript (include quotes in quotation marks "like this" to indicate direct quotes from your manuscript), or elaborate on this item by providing additional information not in the ms, or briefly explain why the item is not applicable/relevant for your study

Yes. These information are clearly indicated on the Consort diagram and tables to the

Your response is too large. Try shortening some answers.

13b-i) Attrition diagram

Strongly recommended: An attrition diagram (e.g., proportion of participants still logging in or using the intervention/comparator in each group plotted over time, similar to a survival curve) or other figures or tables demonstrating usage/dose/engagement.

	1	2	3	4	5	
subitem not at all important	○	○	○	○	◉	essential

Clear selection

Does your paper address subitem 13b-i?

Copy and paste relevant sections from the manuscript or cite the figure number if applicable (include quotes in quotation marks "like this" to indicate direct quotes from your manuscript), or elaborate on this item by providing additional information not in the ms, or briefly explain why the item is not applicable/relevant for your study

Yes. Attrition information is included in the Consort diagram attached to the submission

14a) Dates defining the periods of recruitment and follow-up

Does your paper address CONSORT subitem 14a? *

Copy and paste relevant sections from the manuscript (include quotes in quotation marks "like this" to indicate direct quotes from your manuscript), or elaborate on this item by providing additional information not in the ms, or briefly explain why the item is not applicable/relevant for your study

Yes.

Your response is too large. Try shortening some answers.

14a-i) Indicate if critical “secular events” fell into the study period

Indicate if critical “secular events” fell into the study period, e.g., significant changes in Internet resources available or “changes in computer hardware or Internet delivery resources”

	1	2	3	4	5	
subitem not at all important	○	○	●	○	○	essential

Clear selection

Does your paper address subitem 14a-i?

Copy and paste relevant sections from the manuscript (include quotes in quotation marks "like this" to indicate direct quotes from your manuscript), or elaborate on this item by providing additional information not in the ms, or briefly explain why the item is not applicable/relevant for your study

Not applicable to our study- no critical secular events fell using study period.

14b) Why the trial ended or was stopped (early)

Does your paper address CONSORT subitem 14b? *

Copy and paste relevant sections from the manuscript (include quotes in quotation marks "like this" to indicate direct quotes from your manuscript), or elaborate on this item by providing additional information not in the ms, or briefly explain why the item is not applicable/relevant for your study

Not applicable. Trial did not end or stop early.

15) A table showing baseline demographic and clinical characteristics for each group

NPT: When applicable, a description of care providers (case volume, qualification, expertise, etc.) and centers (volume) in each group

Your response is too large. Try shortening some answers.

Does your paper address CONSORT subitem 15? *

Copy and paste relevant sections from the manuscript (include quotes in quotation marks "like this" to indicate direct quotes from your manuscript), or elaborate on this item by providing additional information not in the ms, or briefly explain why the item is not applicable/relevant for your study

Yes.

15-i) Report demographics associated with digital divide issues

In ehealth trials it is particularly important to report demographics associated with digital divide issues, such as age, education, gender, social-economic status, computer/Internet/ehealth literacy of the participants, if known.

	1	2	3	4	5	
subitem not at all important	○	○	●	○	○	essential

Clear selection

Does your paper address subitem 15-i? *

Copy and paste relevant sections from the manuscript (include quotes in quotation marks "like this" to indicate direct quotes from your manuscript), or elaborate on this item by providing additional information not in the ms, or briefly explain why the item is not applicable/relevant for your study

Yes. Participants' age and gender are included in the tables in the results segment

16) For each group, number of participants (denominator) included in each analysis and whether the analysis was by original assigned groups

Your response is too large. Try shortening some answers.

16-i) Report multiple “denominators” and provide definitions

Report multiple “denominators” and provide definitions: Report N’s (and effect sizes) “across a range of study participation [and use] thresholds” [1], e.g., N exposed, N consented, N used more than x times, N used more than y weeks, N participants “used” the intervention/comparator at specific pre-defined time points of interest (in absolute and relative numbers per group). Always clearly define “use” of the intervention.

	1	2	3	4	5	
subitem not at all important	○	○	○	○	◉	essential

Clear selection

Does your paper address subitem 16-i? *

Copy and paste relevant sections from the manuscript (include quotes in quotation marks "like this" to indicate direct quotes from your manuscript), or elaborate on this item by providing additional information not in the ms, or briefly explain why the item is not applicable/relevant for your study

Our app program runs for 9-days with clear instructions of use for all participants. Segment on "Body Image Program" and "Cooperation Learning Program" define each of the program usage clearly.

16-ii) Primary analysis should be intent-to-treat

Primary analysis should be intent-to-treat, secondary analyses could include comparing only “users”, with the appropriate caveats that this is no longer a randomized sample (see 18-i).

	1	2	3	4	5	
subitem not at all important	○	○	○	○	◉	essential

Clear selection

Your response is too large. Try shortening some answers.

Does your paper address subitem 16-ii?

Copy and paste relevant sections from the manuscript (include quotes in quotation marks "like this" to indicate direct quotes from your manuscript), or elaborate on this item by providing additional information not in the ms, or briefly explain why the item is not applicable/relevant for your study

Yes. "Intent-to-treat analyses were conducted for participants who were lost to follow-up by carrying forward their last reported scores. Missing scores for AES were substituted with the mean score. Independent t tests did not reveal any attrition-related biases across demographic and outcome variables (P>.10)."

17a) For each primary and secondary outcome, results for each group, and the estimated effect size and its precision (such as 95% confidence interval)

Does your paper address CONSORT subitem 17a? *

Copy and paste relevant sections from the manuscript (include quotes in quotation marks "like this" to indicate direct quotes from your manuscript), or elaborate on this item by providing additional information not in the ms, or briefly explain why the item is not applicable/relevant for your study

Yes. Effect sizes of the main analyses outcomes are included in tables 6 and 7.

17a-i) Presentation of process outcomes such as metrics of use and intensity of use

In addition to primary/secondary (clinical) outcomes, the presentation of process outcomes such as metrics of use and intensity of use (dose, exposure) and their operational definitions is critical. This does not only refer to metrics of attrition (13-b) (often a binary variable), but also to more continuous exposure metrics such as “average session length”. These must be accompanied by a technical description how a metric like a “session” is defined (e.g., timeout after idle time) [1] (report under item 6a).

	1	2	3	4	5	
subitem not at all important	○	○	◉	○	○	essential

Clear selection

Your response is too large. Try shortening some answers.

Does your paper address subitem 17a-i?

Copy and paste relevant sections from the manuscript (include quotes in quotation marks "like this" to indicate direct quotes from your manuscript), or elaborate on this item by providing additional information not in the ms, or briefly explain why the item is not applicable/relevant for your study

Not applicable. Process outcomes were not used in this study.

17b) For binary outcomes, presentation of both absolute and relative effect sizes is recommended

Does your paper address CONSORT subitem 17b? *

Copy and paste relevant sections from the manuscript (include quotes in quotation marks "like this" to indicate direct quotes from your manuscript), or elaborate on this item by providing additional information not in the ms, or briefly explain why the item is not applicable/relevant for your study

Not applicable. Outcomes are not binary in this study.

18) Results of any other analyses performed, including subgroup analyses and adjusted analyses, distinguishing pre-specified from exploratory

Does your paper address CONSORT subitem 18? *

Copy and paste relevant sections from the manuscript (include quotes in quotation marks "like this" to indicate direct quotes from your manuscript), or elaborate on this item by providing additional information not in the ms, or briefly explain why the item is not applicable/relevant for your study

Not applicable- no other analyses are performed.

Your response is too large. Try shortening some answers.

18-i) Subgroup analysis of comparing only users

A subgroup analysis of comparing only users is not uncommon in ehealth trials, but if done, it must be stressed that this is a self-selected sample and no longer an unbiased sample from a randomized trial (see 16-iii).

	1	2	3	4	5	
subitem not at all important	○	○	◉	○	○	essential

Clear selection

Does your paper address subitem 18-i?

Copy and paste relevant sections from the manuscript (include quotes in quotation marks "like this" to indicate direct quotes from your manuscript), or elaborate on this item by providing additional information not in the ms, or briefly explain why the item is not applicable/relevant for your study

Not applicable. Subgroup analysis of comparing only users not conducted.

19) All important harms or unintended effects in each group

(for specific guidance see CONSORT for harms)

Does your paper address CONSORT subitem 19? *

Copy and paste relevant sections from the manuscript (include quotes in quotation marks "like this" to indicate direct quotes from your manuscript), or elaborate on this item by providing additional information not in the ms, or briefly explain why the item is not applicable/relevant for your study

There are no foreseeable discomforts and risks for participants in our study.

Your response is too large. Try shortening some answers.

19-i) Include privacy breaches, technical problems

Include privacy breaches, technical problems. This does not only include physical “harm” to participants, but also incidents such as perceived or real privacy breaches [1], technical problems, and other unexpected/unintended incidents. “Unintended effects” also includes unintended positive effects [2].

	1	2	3	4	5	
subitem not at all important	○	○	○	○	◉	essential

Clear selection

Does your paper address subitem 19-i?

Copy and paste relevant sections from the manuscript (include quotes in quotation marks "like this" to indicate direct quotes from your manuscript), or elaborate on this item by providing additional information not in the ms, or briefly explain why the item is not applicable/relevant for your study

The participant information sheet that participants read and consented as part of the study (segment on "Procedure and Participant Flow" in the paper) addressed privacy and confidentiality concerns, as well as who to contact should they require assistance.

19-ii) Include qualitative feedback from participants or observations from staff/researchers

Include qualitative feedback from participants or observations from staff/researchers, if available, on strengths and shortcomings of the application, especially if they point to unintended/unexpected effects or uses. This includes (if available) reasons for why people did or did not use the application as intended by the developers.

	1	2	3	4	5	
subitem not at all important	○	○	◉	○	○	essential

Clear selection

Your response is too large. Try shortening some answers.

Does your paper address subitem 19-ii?

Copy and paste relevant sections from the manuscript (include quotes in quotation marks "like this" to indicate direct quotes from your manuscript), or elaborate on this item by providing additional information not in the ms, or briefly explain why the item is not applicable/relevant for your study

Not applicable. Qualitative feedback was not obtained in our study.

DISCUSSION

22) Interpretation consistent with results, balancing benefits and harms, and considering other relevant evidence

NPT: In addition, take into account the choice of the comparator, lack of or partial blinding, and unequal expertise of care providers or centers in each group

22-i) Restate study questions and summarize the answers suggested by the data, starting with primary outcomes and process outcomes (use)

Restate study questions and summarize the answers suggested by the data, starting with primary outcomes and process outcomes (use).

	1	2	3	4	5	
subitem not at all important	○	○	○	○	◉	essential

Clear selection

Does your paper address subitem 22-i? *

Copy and paste relevant sections from the manuscript (include quotes in quotation marks "like this" to indicate direct quotes from your manuscript), or elaborate on this item by providing additional information not in the ms, or briefly explain why the item is not applicable/relevant for your study

Yes. "Discussion" segment of the paper described these information.

Your response is too large. Try shortening some answers.

22-ii) Highlight unanswered new questions, suggest future research

Highlight unanswered new questions, suggest future research.

	1	2	3	4	5	
subitem not at all important	○	○	○	○	◉	essential

Clear selection

Does your paper address subitem 22-ii?

Copy and paste relevant sections from the manuscript (include quotes in quotation marks "like this" to indicate direct quotes from your manuscript), or elaborate on this item by providing additional information not in the ms, or briefly explain why the item is not applicable/relevant for your study

Yes. "As literature in this area remains relatively new, further research is required to improve our understanding of gender role norms in self-compassion, to effectively tailor self-compassion approaches for female and male participants.", "To capture male participants' body image concerns more accurately, male-specific measures such as Male Body Attitudes Scale [90] can be used in future studies.", "Finally, in light of increasing studies which found self-compassion to mediate the effects of body image interventions, future studies can examine self-compassion as a mechanism of change. It would also be beneficial to obtain qualitative feedback from participants on elements of the body image program, such as their perception of the tone or number of intervention messages, to evaluate the effectiveness of the program."

20) Trial limitations, addressing sources of potential bias, imprecision, and, if relevant, multiplicity of analyses

20-i) Typical limitations in ehealth trials

Typical limitations in ehealth trials: Participants in ehealth trials are rarely blinded. Ehealth trials often look at a multiplicity of outcomes, increasing risk for a Type I error. Discuss biases due to non-use of the intervention/usability issues, biases through informed consent procedures, unexpected events.

	1	2	3	4	5	
subitem not at all important	○	○	○	○	◉	essential

Clear selection

Your response is too large. Try shortening some answers.

Does your paper address subitem 20-i? *

Copy and paste relevant sections from the manuscript (include quotes in quotation marks "like this" to indicate direct quotes from your manuscript), or elaborate on this item by providing additional information not in the ms, or briefly explain why the item is not applicable/relevant for your study

Yes. "This study has some limitations. First, student participants may limit the generalizability of findings, as university students and the public may differ in factors such as level of education [89]. Hence, the university sample may not be representative of the general young adult population. Secondly, self-report measures are prone to social desirability bias, expectancies, and demand characteristics, which all may have contributed to the observed effects. Third, incentivizing participants with course credits or money may have motivated their participation and retention.

The sample size for male participants in our study was small despite additional recruitment efforts and thus was likely underpowered. Moreover, in view of sex differences in body image, more research is required to better understand ways to increase the effectiveness of mobile-based body image programs for male participants in a mixed-sex format and address muscularity concerns. "

21) Generalisability (external validity, applicability) of the trial findings

NPT: External validity of the trial findings according to the intervention, comparators, patients, and care providers or centers involved in the trial

21-i) Generalizability to other populations

Generalizability to other populations: In particular, discuss generalizability to a general Internet population, outside of a RCT setting, and general patient population, including applicability of the study results for other organizations

	1	2	3	4	5	
subitem not at all important	○	○	○	○	◉	essential

Clear selection

Your response is too large. Try shortening some answers.

Does your paper address subitem 21-i?

Copy and paste relevant sections from the manuscript (include quotes in quotation marks "like this" to indicate direct quotes from your manuscript), or elaborate on this item by providing additional information not in the ms, or briefly explain why the item is not applicable/relevant for your study

Yes. "Future research should seek to further enhance the program's effectiveness with the wider young adult population."

21-ii) Discuss if there were elements in the RCT that would be different in a routine application setting

Discuss if there were elements in the RCT that would be different in a routine application setting (e.g., prompts/reminders, more human involvement, training sessions or other co-interventions) and what impact the omission of these elements could have on use, adoption, or outcomes if the intervention is applied outside of a RCT setting.

	1	2	3	4	5	
subitem not at all important	○	○	◉	○	○	essential

Clear selection

Does your paper address subitem 21-ii?

Copy and paste relevant sections from the manuscript (include quotes in quotation marks "like this" to indicate direct quotes from your manuscript), or elaborate on this item by providing additional information not in the ms, or briefly explain why the item is not applicable/relevant for your study

No applicable for our study.

OTHER INFORMATION

23) Registration number and name of trial registry

Your response is too large. Try shortening some answers.

Does your paper address CONSORT subitem 23? *

Copy and paste relevant sections from the manuscript (include quotes in quotation marks "like this" to indicate direct quotes from your manuscript), or elaborate on this item by providing additional information not in the ms, or briefly explain why the item is not applicable/relevant for your study

Trial Registration: ClinicalTrials.gov NCT04977973

24) Where the full trial protocol can be accessed, if available

Does your paper address CONSORT subitem 24? *

Cite a Multimedia Appendix, other reference, or copy and paste relevant sections from the manuscript (include quotes in quotation marks "like this" to indicate direct quotes from your manuscript), or elaborate on this item by providing additional information not in the ms, or briefly explain why the item is not applicable/relevant for your study

Not applicable. The authors of this study can be contacted for further queries pertaining to this study.

25) Sources of funding and other support (such as supply of drugs), role of funders

Does your paper address CONSORT subitem 25? *

Copy and paste relevant sections from the manuscript (include quotes in quotation marks "like this" to indicate direct quotes from your manuscript), or elaborate on this item by providing additional information not in the ms, or briefly explain why the item is not applicable/relevant for your study

Yes. "The study was partly funded by Intellect Pte Ltd. The study design, data management, interpretation, analysis, and reporting and the decision to publish the study are entirely independent of Intellect Pte Ltd."

X27) Conflicts of Interest (not a CONSORT item)

Your response is too large. Try shortening some answers.

X27-i) State the relation of the study team towards the system being evaluated

In addition to the usual declaration of interests (financial or otherwise), also state the relation of the study team towards the system being evaluated, i.e., state if the authors/evaluators are distinct from or identical with the developers/sponsors of the intervention.

	1	2	3	4	5	
subitem not at all important	○	○	○	○	◉	essential

Clear selection

Does your paper address subitem X27-i?

Copy and paste relevant sections from the manuscript (include quotes in quotation marks "like this" to indicate direct quotes from your manuscript), or elaborate on this item by providing additional information not in the ms, or briefly explain why the item is not applicable/relevant for your study

Yes. "Conflicts of Interest
The study was partly funded by Intellect Pte Ltd. The study design, data management, interpretation, analysis, and reporting and the decision to publish the study are entirely independent of Intellect Pte Ltd. OS had a research collaboration with Intellect Pte Ltd at the time of the data collection and has since joined Intellect Pte Ltd as their clinical director."

About the CONSORT EHEALTH checklist

As a result of using this checklist, did you make changes in your manuscript? *

- ○ yes, major changes
- ◉ yes, minor changes
- ○ no

What were the most important changes you made as a result of using this checklist?

Your answer

Your response is too large. Try shortening some answers.

How much time did you spend on going through the checklist INCLUDING making changes in your manuscript *

More than 4 hours were spent

As a result of using this checklist, do you think your manuscript has improved? *

- (selected) yes
- no
- Other:

Would you like to become involved in the CONSORT EHEALTH group?

This would involve for example becoming involved in participating in a workshop and writing an "Explanation and Elaboration" document

- yes
- (selected) no
- Other:

Clear selection

Any other comments or questions on CONSORT EHEALTH

Your answer

STOP - Save this form as PDF before you click submit

To generate a record that you filled in this form, we recommend to generate a PDF of this page (on a Mac, simply select "print" and then select "print as PDF") before you submit it.

When you submit your (revised) paper to JMIR, please upload the PDF as supplementary file.

Don't worry if some text in the textboxes is cut off, as we still have the complete information in our database. Thank you!

Your response is too large. Try shortening some answers.

Final step: Click submit !
Click submit so we have your answers in our database!

Submit

Clear form

Never submit passwords through Google Forms.

This content is neither created nor endorsed by Google. Report Abuse - Terms of Service - Privacy Policy

Google Forms

Your response is too large. Try shortening some answers.
